# The Disease-Associated Chaperone FKBP51 Impairs Cognitive Function by Accelerating AMPA Receptor Recycling

**DOI:** 10.1523/ENEURO.0242-18.2019

**Published:** 2019-03-01

**Authors:** Laura J. Blair, Marangelie Criado-Marrero, Dali Zheng, Xinming Wang, Siddharth Kamath, Bryce A. Nordhues, Edwin J. Weeber, Chad A. Dickey

**Affiliations:** 1 Department of Molecular Medicine, University of South Florida, Byrd Institute, Tampa, FL 33613; 2 Department of Pharmacology and Physiology, University of South Florida, Byrd Institute, Tampa, FL 33613

**Keywords:** AMPA receptor, chaperone, FKBP5, Hsp90

## Abstract

Increased expression of the FK506-binding protein 5 (*FKBP5*) gene has been associated with a number of diseases, but most prominently in connection to psychiatric illnesses. Many of these psychiatric disorders present with dementia and other cognitive deficits, but a direct connection between these issues and alterations in *FKBP5* remains unclear. We generated a novel transgenic mouse to selectively overexpress *FKBP5,* which encodes the FKBP51 protein, in the corticolimbic system, which had no overt effects on gross body weight, motor ability, or general anxiety. Instead, we found that overexpression of FKBP51 impaired long-term depression (LTD) as well as spatial reversal learning and memory, suggesting a role in glutamate receptor regulation. Indeed, FKBP51 altered the association of heat-shock protein 90 (Hsp90) with AMPA receptors, which was accompanied by an accelerated rate of AMPA recycling. In this way, the chaperone system is critical in triage decisions for AMPA receptor trafficking. Imbalance in the chaperone system may manifest in impairments in both inhibitory learning and cognitive function. These findings uncover an unexpected and essential mechanism for learning and memory that is controlled by the psychiatric risk factor *FKBP5*.

## Significance Statement

Candidate studies have identified a link between FK506-binding protein 5 (*FKBP5*) allelic variants and psychiatric disorders. Patients with these disorders often present with cognitive defects. *FKBP5* variants have been correlated to decreased hippocampal volume, but a direct connection between *FKBP5* and cognitive function has not been established. Here, we have found that mice with high levels of *FKBP5* have altered reversal learning and memory, which may be through direct regulation of neuronal activity by regulating AMPA receptors.

## Introduction

The 51-kDa FK506-binding protein (*FKBP5*/FKBP51) has been genetically linked to a number of disorders associated with affective symptoms as well as impairments in learning and memory. *FKBP5* expression can be enhanced by stress as well as common single nucleotide polymorphisms (SNPs; Klengel et al., 2013). These SNPs can increase suceptibility for post-traumatic stress disorder (PTSD; Klengel et al., 2013), major depression (Binder et al., 2004; Klengel et al., 2013), cognitive decline in aging (Fujii et al., 2014), suicide, aggression, and violent behavior (Binder et al., 2008; Bevilacqua et al., 2012). Moreover, *FKBP5* expression progressively increases with normal aging, concomitant with reduced *FKBP5* DNA methylation, and has been associated with the number one cause of dementia in the world, Alzheimer’s disease (Jinwal et al., 2010; Blair et al., 2013; Sabbagh et al., 2014).

While some viral-mediated overexpression studies have attempted to model increased *FKBP5* levels in the murine central nervous system, no stable transgenic models have been made. These viral studies have demonstrated that *FKBP5* overexpression in the amygdala, but not the hippocampus, can increase anxiety-related behavior (Hartmann et al., 2015). However, conversely, viral-mediated knock-down of *Fkbp5* in the amygdala prevented stress-induced fear (Attwood et al., 2011). Moreover, mice lacking the *Fkbp5* gene show reduced hypothalamic-pituitary-adrenal (HPA) axis reactivity and protection from stress-induced (Touma et al., 2011; Hartmann et al., 2012) and age-induced (O'Leary et al., 2011) depressive-like behavior. These studies have highlighted potential roles for *FKBP5/*FKBP51 in aging, cognition, and disease. But a model to investigate the consequences of increased *FKBP5* expression in the corticolimbic system has not been developed despite some of the clinical phenotypes clearly being related to learning and memory.

Here, we filled this gap by generating the first transgenic mouse to overexpress FKBP51 throughout the forebrain, beginning early in the post-natal period as determined by the expression pattern of CamKIIα. FKBP51 overexpression had a pronounced, and previously unknown, effect on inhibitory learning and plasticity through altered AMPA receptor dynamics. Specifically, we identified that high levels of FKBP51 accelerated the recycling of internalized AMPA receptors back to the synaptic membrane. This may be due to direct interaction of FKBP51 with heat-shock protein 90 (Hsp90) while in complex with GluR1-type AMPA receptors, as our data suggests. In addition to allowing us to identify a critical mechanism that regulates fate decisions for GluR1-type AMPA receptors, these novel *FKBP5*-expressing mice could also be used in future studies to assess other FKBP51-related phenotypes, particularly using stress or gene × environment paradigms, as well as to test the efficacy of emerging therapeutics designed to target FKBP51.

## Materials and Methods

### Antibodies and reagents

pBT378 and pTR-tTA-GFP were a kind gift from Noreen Luetteke. pCI-SEP-GluR1 (plasmid #24000) was purchased from Addgene. Human *FKBP5* (Hs01551006_m1), Mouse *Fkbp5* (mm00487406_g1), and mouse *Gapdh* (mm99999915_g1) Real-time PCR TaqMan probes were purchased from Applied Biosystems and used in combination with an RNA-to-C_T_ *1-Step* kit (Applied Biosystems). All Antibodies used at 1:1000 unless otherwise indicated. The following antibodies were used: α-GAPDH (Meridian Life Science catalog #H86045M, RRID:AB_497737) and α-FKBP51 (clone Hi51B) and α-FKBP51 (clone Hi51E) antibodies were a kind gift from Marc Cox. Hi51B is the same clone that can be commercially purchased (StressMarq Biosciences catalog #SMC-138, RRID:AB_2570356, Novus catalog #NB110-96873, RRID:AB_1260804, or Abcam catalog #ab79844, RRID:AB_2103132). Clone Hi51E has been previously characterized (Nair et al., 1997; Gross et al., 2008). α-GluR1 (Cell Signaling Technology catalog #13185, RRID:AB_2732897), α-glucocorticoid receptor (GR; Cell Signaling Technology catalog #3660S, RRID:AB_11179215), biotin-conjugated α-NeuN (Millipore catalog #MAB377B, RRID:AB_177621), and α-Hsp90 (StressMarq Biosciences catalog #SMC-149, RRID:AB_2570363). The following HRP-tagged α-mouse and α-rabbit secondary antibodies were used for Western blottings (SouthernBiotech catalog #1030-05, RRID:AB_2619742 and SouthernBiotech catalog #4050-08, RRID:AB_2732896). The following BIOT-labeled α-mouse secondary antibody (SouthernBiotech catalog #1020-08, RRID:AB_2737411) was used for immunohistochemical staining. *S*-AMPA and NMDA were purchased from Tocris Bioscience.

### Construction of the rTgFKBP5 vector

The responder transgene was generated by two subsequent subcloning steps. First, following the addition of a PmeI site by mutagenesis, we ligated human *FKBP5* cDNA into Tet-Op-mp1 pA vector through digestion of the ClaI and EcoRV cut sites. This plasmid, TetO-FKBP5, was subcloned into pBT378 through digestion by PmeI and NotI restrictions sites, which generated the final TgFKBP5 vector. This final vector (TgFKBP5) was used to generate site-specific *FKBP5* transgenic founder mice on the FVB background. This was achieved, based on an established method (Tasic et al., 2011), by co-microinjection of the constructed TgFKBP5 and ΦC31 Integrase into H11P3 homozygous oocytes from fertilized FVB/N mice which have attP insertion sites at the Hipp11 (H11) locus on chromosome 11 by the Stanford Transgenic Research Facility at Stanford University School of Medicine in Stanford, CA.

### Generation and genotyping of rTgFKBP5 mice

Founder mice contained a single copy of *FKBP5* which was inserted into the Hipp11 (H11) locus by irreversible recombination using ΦC31 integrase with complimentary attB and attP sites (Tasic et al., 2011). High expression of this single copy of FKBP5 was generated by crossing TgFKBP5 single-transgenic founder mice on the FVB background with CAMKIIα-tTA mice on the 129S6 background (The Jackson Laboratory; stock 003010). Female FVB FKBP5 mice were bred with male CamKIIα-tTA mice. The offspring of this cross generated the rTgFKBP5 double-transgenic line, which are used for experiments only and not for further breeding. The founding strain is maintained by ordering FVB/NJ female mice (The Jackson Laboratory) to breed with male FKBP5 mice and the CamKIIα-tTA strain is maintained in-house by ordering 129S6/SVEV female (Taconic) mice to breed with male CamKIIα-tTA mice. Genotyping was performed using ear clip digests using the following primers: for the inserted human *FKBP5* gene (F, 5’GTGTACGGTGGGAGGCCTAT3’, and R, 5’GTCCCATGCCTTGATGACTT3’) and CamKIIα-tTA (F, 5′-CGCTGTGGGGCATTTTACTTT-3′, and R,5′-CATGTCCAGATCGAAATGGTC-3′ as well as for the housekeeping gene T-cell receptor δ chain (Tcrd; F, 5′-CAAATGTTGCTTGTCTGGTG-3′, and R, 5′-GTCAGTCGAGTGCACAGTTT-3′) to confirm presence of tissue. DNA was amplified in a ThermoCycler (Thermo Scientific) with the following cycles: 15 min at 95°C, 20× 15 s at 95°C, 30 s at 68°C, 45 s at 72°C, and 10 min at 72°C.The PCR product was then analyzed on a Quiaxcel (QIAGEN). Mice positive for both human *FKBP5* and CamKIIα-tTA were classified as rTgFKBP5. Those positive for the CamKIIα-tTA and lacking the human *FKBP5* gene were classified as tTA, and mice lacking both genes were classified as wild type (WT).

### Tissue collection, brightfield staining, and microscopy

All animal studies were approved and conducted following the guidelines set by the University of South Florida Institutional Animal Care and Use Committee in accordance with the Association for Assessment and Accreditation of Laboratory Animal Care international regulation. Both sexes of mice were used throughout all of the experiments. Body weight was measured multiple times over the lifespan of the mice by self-calibrating scale (Ohaus). For tissue collection and processing, mice were overdosed with pentobarbital solution and transcardially perfused with 0.9% saline solution; brains were rapidly removed and the left hemisphere was placed in 4% paraformaldehyde overnight and the right hemisphere was snap frozen for Western blottings and RNA extraction. Free-floating sections were used for immunohistochemical analysis using 1:100 FKBP51 (Hi51B) antibody Extended data Fig. 2-1. Brightfield stained tissue was imaged by a Zeiss AxioScan.Z1 slide scanner using a 20× objective.

**Figure 1.**
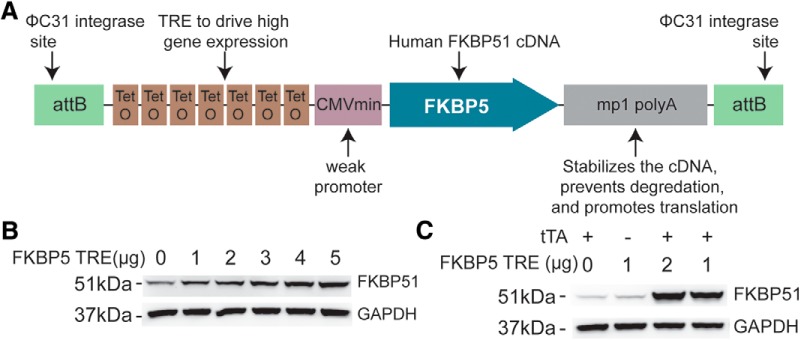
Detailed schematic and validation of the FKBP5 TRE transgene. ***A***, To allow for site-directed, single copy insertion into the mouse genome in chromosome 11, the transgenic construct contained flanking attB sites via a PhiC31 integrase. The downstream Mp1 poly A tail will help maintain stable expression. To drive high expression, the transgenic construct included a tetracycline-response element (TRE) promoter made of seven repeats of the tetracycline operators used to drive high expression of the singly inserted *FKBP5* gene in the presence of the tTA, and a weak minimal CMV promoter which produces low basal expression. ***B***, Western blotting from HEK293T cells transfected with increasing amounts of FKBP5 TRE plasmid, as indicated, for 48 h. ***C***, HEK293T cells were transfected with the indicated amounts of FKBP5 TRE and tTA plasmid, to ensure the tTA would drive high FKBP51 expression.

**Figure 2.**
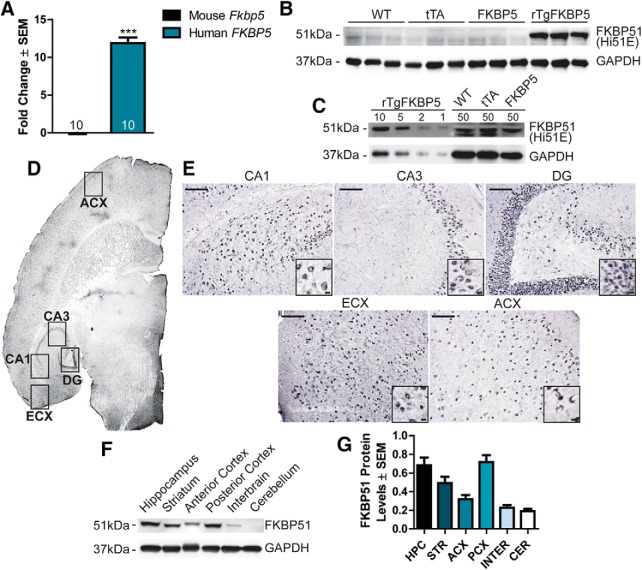
FKBP51 expression and distribution in rTgFKBP5 mice. ***A***, Expression of human and mouse *FKBP5* in rTgFKBP5 mice expressed as fold change ± SEM compared to WT mice using qPCR; ***p < 0.001 by *t* test (*N* = 10) with three technical replicates. ***B***, Western blotting showing FKBP51 levels in the hippocampus from rTgFKBP5, WT, FKBP5, and tTA mice. ***C***, Western blotting showing levels of FKBP51 levels in the rTgFKBP5 hippocampus from 1 to 10 µg of protein loaded compared to 50 µg of protein from WT or FKBP51 mice. GAPDH levels are shown to confirm protein load. See Extended Data Figure 2-1 for more information on the antibody. ***D***, 20× images of anti-FKBP51 staining from rTgFKBP5 mice. The entorhinal cortex (ECX), anterior cortex (ACX), CA1, CA3, and dentate gyrus (DG) are labeled. ***E***, 20× images of anti-FKBP51 staining from rTgFKBP5 mice in the CA1, CA3, DG, ECX, and ACX. Scale bar = 100 µm; 10 µm (inset). ***F***, Western blotting showing FKBP51 levels in the hippocampus, striatum, ACX, posterior cortex (PCX), interbrain, thalamus and hypothalamus, and cerebellum of a rTgFKBP5 mouse. ***G***, Quantitation of FKBP51 proteins levels throughout the hippocampus (HPC), striatum (STR), ACX, PCX, interbrain, thalamus and hypothalamus (INTER), and cerebellum (CER), of rTgFKBP5 mice from multiple exposures.

10.1523/ENEURO.0242-18.2019.f2-1 Extended Data Figure 2-1 FKBP51 (Hi51E) antibody recognizes mouse and human FKBP51 in tissue and mouse cell lysates. *A*, Western blotting of 30 µg of tissue from the hippocampi of two 12-month-old WT mice, tissue from the medial temporal gyrus an 87-year-old male (provided deidentified by the UCI Brain Bank), and tissue from the hippocampus of a one-month-old rTgFKBP5 mouse probed for FKBP51 (Hi51E) and GAPDH antibodies. ***B***, Western blottings from independent experiments using mouse hippocampal HT-22 cells cultured in complete MEM media treated with dexamethasone (Dex) as indicated, to induce FKBP51 expression, probed by FKBP51 (Hi51E) and GAPDH antibodies. Download Figure 2-1, TIF file.

### Western blottings

For Western blottings, mouse tissue was weighed and homogenized by sonication in RIPA buffer (50 mM Tris-HCl, 150 mM NaCl, 2 mM EDTA, 0.1% SDS, 0.5% sodium deoxycholate, and 1% Triton X-100; pH 7.4), except where indicated. A total of 10 µl of buffer was added per 1 mg of brain tissue. Equal amounts of protein, except where indicated, were run on SDS-PAGE gels (Bio-Rad) and processed by Western blotting.

### Real-time quantitative RT-PCR

Frozen mouse brain cortex (∼30 mg) was placed into 1 ml TRIzol (Thermo Scientific) and was homogenized. Briefly, to each 1 ml of TRIzol reagent, 200-µl chloroform was added, mixed vigorously and incubated for 5 min at room temperature before centrifugation at 12,000 × *g* (4°C for 15 min). The upper aqueous layer was separated and added to 2.5 volumes of 100% ethanol. Incubated at –20°C for 1 h following centrifugation at 12,000 × *g* (4°C for 15 min). The supernatant was discarded and the pellet was washed with 1 ml 75% ethanol following centrifugation at 10,000 × *g* (4°C for 15 min). The supernatant was discarded and the pellet was dried carefully. The pellet was resuspended in RNase-free water. To get rid of genomic DNA contamination, total RNA was incubated with RNase-free DNaseI on RNeasy spin columns (QIAGEN) for 15 min and eluted using RNase-free water and measured on Nanodrop 2000 (Thermo Scientific).

The real-time RT-PCR was done using TaqMan RNA-to C_T_ *1-Step* kit (Thermo Scientific) and GeneAmp PCR system 9600 (Applied Biosystem) using manufacturer’s instructions. TaqMan gene expression assays for mouse GAPDH, FKBP51, and human FKBP51 were used. Briefly, 20-µl reaction/tube contained TaqMan RT-PCR Mix (2×), TaqMan Gene Expression Assay (20×), TaqMan RT Enzyme Mix (40×), and Total RNA (50 ng) plus RNase-free water. The thermal cycling conditions were 48°C for 15 min (RT step) and for PCR, 95°C for 10 min, 40 cycles (95°C for 15 s, 60°C for 1 min).

Samples were assayed in a minimum of three replicates and the data were analyzed using the ΔΔC_T_ method (Livak and Schmittgen, 2001). The expression values were normalized to endogenous mouse *Gapdh* control values.

### Rotarod and open field

Motor function was measured by rotarod and open field. For rotarod, three-month-old rTgFKBP5 (*N* = 16; eight males and eight females), WT (*N* = 22; 10 males and 12 females), and tTA control (*N* = 20; 11 males and nine females) mice were placed onto Rota-Rod apparatus (Stoelting; AugoBasile Apparatus) while the rod was rotating at four rotations per minute (rpm). The rod was accelerated at a steady rate to 40 rpm over 300 s. Latency to fall from the rod was measured. For analysis, both sexes were combined, since sex did not have a significant effect on result, see Extended Data Table 1-1 for additional statistical analysis.

Extended Data Table 1-1 Summary of statistical analyses by sex. Download Table 1-1, DOC file.

For open field, three-month-old rTgFKBP5 (*N* = 16; eight males and eight females), WT (*N* = 22; 10 males and 12 females), and tTA control (*N* = 20; 11 males and nine females) mice were monitored for 15 min in an open field (Stoelting). One video for a female tTA mouse did not record properly, so our final tTA *N* = 19. Activity was recorded and analyzed using ANY-maze video tracking software (Stoelting). For analysis, both sexes were combined, since sex did not have a significant effect on result, see Extended Data Table 1-1 for additional statistical analysis.

### Tail suspension test (TST) and Porsolt forced swim test (FST)

For TST and FST, three-month-old old rTgFKBP5 (*N* = 16; eight males and eight females), WT (*N* = 22; 10 males and 12 females), and tTA control (*N* = 20; 11 males and nine females) mice were suspended form their tail for 6 min in a tail-suspension apparatus (Stoelting). Amount of time immobile was recorded. For FST, old rTgFKBP5 (*N* = 16; eight males and eight females), WT (*N* = 22; 10 males and 12 females), and tTA control (*N* = 20; 11 males and nine females) mice were placed in 4L clear, glass cylinders filled to 12 cm with room temperature water and allowed to swim for 6 min. Amount of time immobile was recorded. For analysis, both sexes were combined, since sex did not have a significant effect on result, see Extended Data Table 1-1 for additional statistical analysis.

### Sucrose preference test

To measure pleasuring seeking behavior or lack thereof, we tested the mice using the sucrose preference test. For this task, individually housed three-month-old old rTgFKBP5 (*N* = 16; eight males and eight females), WT (*N* = 22; 10 males and 12 females), and tTA control (*N* = 20; 11 males and nine females) mice were given two water bottles, one filled with tap water and the other filled with 1% sucrose in tap water, which were switched in location after 24 h to help prevent a side preference bias. Following a 48-h habituation, all water was removed for 12 h during the whole dark cycle. Soon after the start of the light cycle, mice were again exposed to the two pre-weighed leak-proof water bottles side-by-side. The positioning of the bottles was randomly assigned across animals to prevent bottle location preference. The bottles were carefully switched in their location at 1 h in each cage and were removed from the cages after 2 h and weighed again. Sucrose preference was determined based on amount of sucrose water consumed compared to the total amount consumed from both bottles combined. Both sexes are shown separately in Extended data Fig. 3-1, since sex had a significant effect, see Extended Data Table 1-1 for additional statistical analysis.

**Figure 3.**
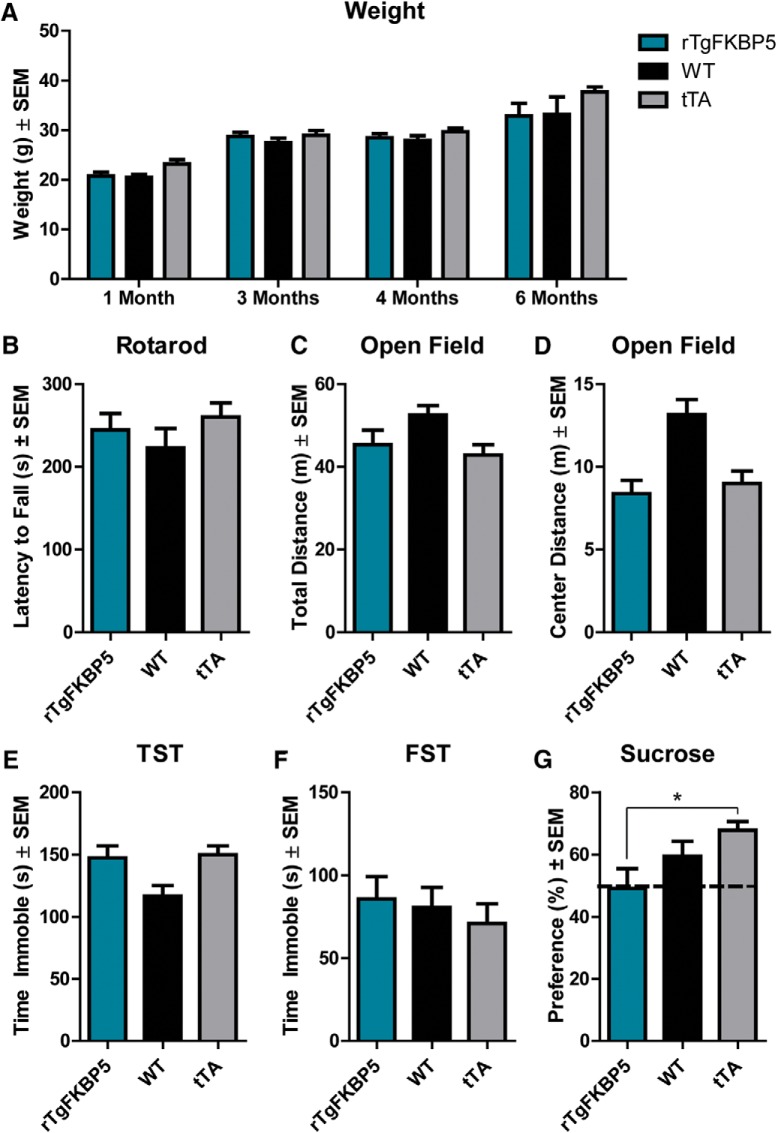
FKBP51 overexpression does not alter basal weight, motor ability, general anxiety, or depression-like behavior but has modest effects on pleasure seeking behavior. ***A***, Whole body weight for rTgFKBP5, WT, and tTA mice was recorded at the indicated time points *N* ≥ 7/genotype at each time point. ***B***, Time on the rotarod apparatus in rTgFKBP5 (*N* = 16), WT (*N* = 22), and tTA (*N* = 20) mice. Total distance traveled (***C***) and distance traveled (***D***) in the center in the open field task was measured for rTgFKBP5 (*N* = 16), WT (*N* = 22), and tTA (*N* = 19) mice. Total immobility time was recorded over 360 s of the (***E***) TST and (***F***) FST. ***G***, Sucrose preference was measured in rTgFKBP5 (*N* = 16), WT (*N* = 22), and tTA (*N* = 20) mice. Consumption was measured by the difference in the weight of bottles filled with sucrose water versus tap water. Sucrose preference percentage ± SEM was determined by the amount (g) of sucrose water consumed versus the amount of total water consumed over the 2-h task. See Extended Data Figure 3-1 for sex differences. *p = 0.0219.

10.1523/ENEURO.0242-18.2019.f3-1 Extended Data Figure 3-1 Anhedonia phenotype in rTgFKBP5 mice is driven by females. Sucrose preference was measured in (***A***) female rTgFKBP5 (*N* = 8), WT (*N* = 10), and tTA (*N* = 11) and (***B***) male rTgFKBP5 (*N* = 8), WT (*N* = 12), and tTA (*N* = 9) mice. Consumption was measured by the difference in the weight of bottles filled with sucrose water versus tap water. Sucrose preference percentage ± SEM was determined by the amount (g) of sucrose water consumed versus the amount of total water consumed over the 2-h task. Download Figure 3-1, TIF file.

### Morris water maze (MWM) and reversal

rTgFKBP5 (*N* = 10; six males and four females), WT (*N* = 10; five males and fivefemales), and tTA control (*N* = 10; five males and five females) mice were trained to locate a hidden escape platform. Each mouse was given 4- to 60-s trials per day with a 1-min intertrial interval in a randomized quadrant. If the target platform was not found, the mice were led to the platform, where they remained for 15 s. The training was completed over 4 d, when the control group was able to find the platform by 10 s. Twenty-four hours later, the hidden platform was removed for the probe test. Mice were placed in the pool in the quadrant opposite of where the target location and allowed to swim for 60 s. The following day, the hidden platform was returned to the pool in the quadrant opposite to the initial training. Mice were retrained to locate this platform until the control group was able to identify the location by 10 s. The next day, mice were subjected to another probe trial. Each day was recorded and analyzed using ANY-maze video tracking software. For analysis, both sexes were combined, since sex did not have a significant effect on result, see Extended Data Table 1-1 for additional statistical analysis.

### Electrophysiology recordings

Acute *ex vivo* hippocampal brain slices were prepared for each of the electrophysiological experiments. Mice of either sex between the ages of 2- and 3.5-months were killed by rapid decapitation and brains were quickly removed and placed into oxygenated, ice-cold cutting solution (100 mM sucrose, 60 mM NaCl, 3 mM KCl, 1.25 mM NaH_2_PO_4_, 28 mM HNaCO_3_, 5 mM D-glucose, 0.6 mM ascorbate, 0.5 mM CaCl_2_, and 7 mM MgCl_2_). Tissue was then sectioned in ice-cold cutting solution with a depth of 400 µm using a vibratome (Leica Biosystems) and hippocampi were carefully excised from the rest of the tissue. Hippocampal tissue was exposed to a 50:50 cutting and artificial CSF (ACSF) solution (125 mM NaCl, 2.5 mM KCl, 1.25 mM NaH_2_PO_4_, 25 mM HNaCO_3_, 25 mM D-glucose, 2 mM CaCl_2_, and 1 mM MgCl_2_) for 10 min. Hippocampi were then allowed to recover in an interface chamber for 1 h in 30°C ACSF. Stimulating and recording electrodes were placed along the Schaffer collateral pathway in the CA3 and CA1, respectively. Slices were then stimulated for 5–10 min, to establish a consistent response to voltage stimulus. Once a consistent response to a voltage stimulus was established, threshold voltage for evoking fEPSPs was determined and the voltage was increased incrementally every 0.5 mV until the maximum amplitude of the fEPSP was reached [input-output (I-O) curve]. All other stimulation paradigms were induced at the same voltage, defined as 50% of the stimulus voltage used to produce the maximum fEPSP amplitude, for each individual slice. This method ensures that recordings made following low-frequency stimulation (LFS) or multiple trains of high-frequency stimulation (HFS) can be compared within groups. Slices were included in analysis if baseline was consistently maintained, as determined by a 10% or less variation in signal.

### Long-term potentiation (LTP)

Following a 20-min baseline recording, slices from either sex were stimulated by either one-train [rTgFKBP5 (*N* = 5; 18 slices), WT (*N* = 6; 29 slices), and tTA (*N* = 5; 22 slices)] or four-trains [rTgFKBP5 (*N* = 7; 16 slices), WT (*N* = 6; 23 slices), and tTA (*N* = 7; 26 slices)] of 100-Hz stimulation for 1 s with a pulse width of 0.100 ms. Evoked potentiation (fEPSPs), as measured by the initial slope normalized to the averaged baseline slope of each slice, was recorded for 60 min following HFS.

### I-O

I-O was recorded in rTgFKBP5 (*N* = 15; 65 slices), WT (*N* = 15; 62 slices), and tTA (*N* = 19; 77 slices) slices from either sex to establish threshold voltage for evoking a fEPSP response. This was achieved by increasing voltage in 0.5mV increments, until maximum amplitude of the fEPSP was reached. Presynaptic I-O was measured by the amplitude of the fiber volley, as a direct read out of depolarization of the presynaptic terminal and the postsynaptic I-O was measured by the initial slope of the fEPSP wave form (Zhang et al., 2009).

### Paired-pulse facilitation (PPF)

As a measurement of short-term plasticity, PPF was recorded in rTgFKBP5 (*N* = 31 mice; 175 slices), WT (*N* = 33; 198 slices), and tTA (*N* = 30; 181 slices) slices from either sex using sequential pulses at 50% of the maximum fEPSP. Recordings were performed over 15–300 ms of interpulse intervals. The data are derived from comparing the slope of the fEPSPs at baseline and following the sequential pulse at 20 ms intervals.

### Long-term depression (LTD)

Acute *ex vivo* slices were prepared as described above, following a 20-min baseline recording, slices from either sex rTgFKBP5 (*N* = 9; 21 slices), WT (*N* = 9; 23 slices), and tTA control (*N* = 10; 31 slices) mice were stimulated by 900 pulses of LFS (3 Hz) to induce LTD. Evoked depotentiation was recorded, as calculated by the slope of the initial fEPSPs normalized to baseline, for 60 min following stimulation.

### Acute *ex vivo* drug dumps AMPA receptor internalization assays

Slices from male rTgFKBP5 or control (WT and tTA combined) mice were prepared as described above for electrophysiological recordings. Following sectioning, slices were allowed to recover for 1 h with Leupeptin, hippocampi were rapidly chilled in ice cold ASCF. Tissue was incubated with 1 mg/ml Sulfo-NHS-SS-biotin for 30 min on a rocker at 4°C. Hippocampi were immediately washed with ice-cold ACSF and placed directly into 20 µM NMDA in 30°C ACSF for 5 min, except for the untreated samples, which were kept in ice-cold ACSF. Hippocampi, except for the 0-min time point which was placed directly into ice cold ACSF, were then transferred to 30°C ACSF and incubated for the indicated times. After each time point elapsed, hippocampi were removed from the solution and placed in ice-cold ACSF and maintained at 4°C for the remainder of the experiment. The biotin remaining on the surface was then cleaved using ice-cold glutathione cleavage solution (50 mM glutathione, 75 mM NaCl, 10 mM EDTA, and 1% BSA; pH 8.75) over two 15-min incubations. The hippocampi were briefly placed into ice-cold glutathione neutralization solution (50 mM iodoacetamide in DPBS, pH7.4). Following two ice-cold ACSF washes, hippocampi were lysed in 200-µl RIPA buffer supplemented with phosphatase inhibitors, protease inhibitors for tissue, and PMSF by sonication. Protein concentrations were determined by BCA, and equal amounts per sample were used for streptavidin co-immunoprecipitation described below.

Streptavidin immunoprecipitation was performed overnight on a rotator using 500 µg of each lysate with 100 µl of washed Streptavidin UltraLink Plus agarose beads (Pierce). The next day, all unbound protein was washed off and the resin was boiled for 5 min in Laemmli buffer with 5% β-mercaptoethanol. The resulting sample was then centrifuged to pellet the beads and the supernatant was then run on SDS-PAGE gels and processed by Western blotting.

### Transfection

HEK293T cells (ATCC catalog #CRL-3216, RRID:CVCL_0063) were transfected using Lipofectamine 2000 (Thermo Scientific), as recommended by the manufacturer, with FKBP5 TRE or control plasmid with or without tTA plasmid for 48 h. Cells were then washed with PBS and harvested in RIPA buffer supplemented with protease and phosphatase inhibitors. Following sonication, cells were incubated on ice for 15 min, cell debris was pelleted by centrifugation at 13,000 × *g* for 15 min, and protein in the supernatant was determined by BCA and analyzed by Western blotting.

HEK293T cells were transfected using Lipofectamine 2000 with GluR1 and FKBP51 or control plasmid for 48 h. AMPA (100 µM, 10 min) was applied just before harvest. Cells were then washed with PBS and harvested in RIPA buffer supplemented with protease and phosphatase inhibitors. Following sonication, cells were incubated on ice and processed as described below.

### GluR1 co-immunoprecipitation

Male rTgFKBP5 and control mice (WT and tTA combined) were overdosed with pentobarbital solution and transcardially perfused with 0.9% saline solution; brains were rapidly removed. The hippocampi were carefully excised and immediately put into ice-cold co-immunoprecipitation buffer (50 mM Tris-HCl, 1% Triton X-100, 1 mM EDTA, and 150 mM NaCl) and sonicated on ice. Tissue lysates were pelleted at 10,000 RCF for 5 min, and the remaining supernatant was pre-cleared with unlabeled-mouse IgG (SouthernBiotech catalog #0107-01, RRID:AB_2732898) and Protein A Dynabeads (Life Technologies) for 90 min. Following pre-clearing and protein estimation, GluR1 antibody was added to 2 mg of protein per sample and rotated overnight at 4°C. Rabbit IgG unlabeled (SouthernBiotech catalog #0111-01, RRID:AB_2732899) was used for IgG control sample. GluR1/control antibody and bound proteins were then isolated using 50 µl of Protein A Dynabeads. Following four washes, the beads/antibody were disassociated from bound proteins by a 5-min incubation at 100°C in 35 µl of 2× Laemmli buffer with 5% β-mercaptoethanol. Beads were isolated using a magnet and the resulting protein sample was processed by Western blotting.

Whole homogenate lysates from HEK293T cells transfected pCI-SEP-GluR1 was a gift from Robert Malinow (Addgene plasmid # 24000 ; RRID:Addgene_24000, Kopec et al., 2006) and either pCMV6 empty vector or pCMV6 FKBP5 ± 100 µM AMPA treatment were precleared with Protein A Dynabeads. Co-immunoprecipitation was performed using 2 mg of precleared lysate incubated with either rabbit IgG UNLB (Southern Biotech) or rabbit α-C-GluR1 (Millipore catalog #AB1504, RRID:AB_2113602) antibody, respectively, overnight on a rocker at 4°C. A total of 50 µl of Protein A magnetic Dynabeads was added to each tube and this mixture was incubated on a rocker at 4°C for 4 h. Following bead conjugation, bound proteins were isolated using a magnet and washed four times. Beads and bound proteins were then disassociated as described above. Tubes were placed on a magnetic strip, and the whole supernatant was processed by Western blotting.

### Statistical analysis

Data were investigated for normality using a D’Agostino and Pearson test and data in graphs and text are presents as the mean ± SEM, unless otherwise stated; *p* values below 0.05 were considered significant. All analysis was performed using GraphPad Prism 5 and is summarized in detail in Table 1. Analysis for sex differences in behavioral tasks is included in Extended Data Table 1-1. To compare two groups, an unpaired two-tailed Student’s *t* test was used for parametric data and the Mann–Whitney test was used for non-parametric analysis. Groups larger than two were evaluated by one-way or two-way ANOVA, as appropriate with genotype and age/quadrant/time as factors. *F* values are reported as decreases of freedom between groups and within groups. Significance is shown in the figures for results where rTgFKBP5 mice are significantly different from control littermates.

**Table 1.** Summary of statistical analyses

Figures	*N*	M and F	Age	Type of test	Factor	Statistical value	*p*
2*A*	rTgFKBP5 (*N* = 10), WT (*N* = 10)	M and F	4–6 months old	Student’s *t* test (unpaired, two-tailed)	Genotype	*F* = 25, DFn = 9	*p* < 0.0001
3*A*	rTgFKBP5 (1M *N* = 16; 3M *N* = 26, 4M *N* = 21; 6M *N* = 7); WT (1M *N* = 19; 3M *N* = 33, 4M *N* = 26; 6M *N* = 6) tTA (1M *N* = 9; 3M *N* = 15, 4M *N* = 22; 6M *N* = 9)	M and F	Varies	Two-way repeated-measures ANOVA with Bonferroni *post hoc* test	Genotype × age	*F* = 0.63; DFn = 6	*p* = 0.705
					Genotype	*F* = 5.44; DFn = 2	*p* = 0.005
					Age	*F* = 48.97; DFn = 3	*p* < 0.0001
3*B*	rTgFKBP5 (*N* = 16), WT (*N* = 22), tTA (*N* = 20)	M and F	3 months old	One-way ANOVA with Tukey *post hoc* test	Genotype	*F* = 0.8867	*p* = 0.4178
3*C*	rTgFKBP5 (*N* = 16), WT (*N* = 22), tTA (*N* = 19)	M and F	3 months old	One-way ANOVA with Tukey *post hoc* test	Genotype	*F* = 3.749	*p* = 0.0299
3*D*	rTgFKBP5 (*N* = 16), WT (*N* = 22), tTA (*N* = 19)	M and F	3 months old	One-way ANOVA with Tukey *post hoc* test	Genotype	*F* = 10.04	*p* = 0.0002
3*E*	rTgFKBP5 (*N* = 16), WT (*N* = 22), tTA (*N* = 20)	M and F	3 months old	One-way ANOVA with Tukey *post hoc* test	Genotype	*F* = 5.099	*p* = 0.0093
3*F*	rTgFKBP5 (*N* = 16), WT (*N* = 22), tTA (*N* = 20)	M and F	3 months old	One-way ANOVA with Tukey *post hoc* test	Genotype	*F* = 0.3405	*p* = 0.7129
3*G*	rTgFKBP5 (*N* = 16), WT (*N* = 22), tTA (*N* = 20)	M and F	3 months old	One-way ANOVA with Tukey *post hoc* test	Genotype	*F* = 4.094	*p* = 0.0219
4*A*	rTgFKBP5 (*N* = 10), WT (*N* = 10), tTA (*N* = 10)	M and F	4–6 months old	Two-way repeated-measures ANOVA with Bonferroni *post hoc* test	Genotype × training day	*F* = 0.26; DFn = 6	*p* = 0.9525
					Genotype	*F* = 7.39; DFn = 2	*p* = 0.0010
					Training day	*F* = 21.56; DFn = 3	*p* < 0.0001
4*B*	rTgFKBP5 (*N* = 10), WT (*N* = 10), tTA (*N* = 10)	M and F	4–6 months old	One-way ANOVA with Tukey *post hoc* test	Quadrant time	rTgFKBP5: *F* = 9.948; WT: *F* = 31.30; tTA: *F* = 15.97	rTgFKBP5: *p* < 0.0001; WT: *p* < 0.0001; tTA: *p* < 0.0001;
4*C*	rTgFKBP5 (*N* = 10), WT (*N* = 10), tTA (*N* = 10)	M and F	4–6 months old	Two-way repeated-measures ANOVA with Bonferroni *post hoc* test	Genotype × training day	*F* = 0.82; DFn = 4	*p* = 0.5146
					Genotype	*F* = 9.49; DFn = 2	*p* = 0.0002
					Training day	*F* = 12.66; DFn = 2	*p* < 0.0001
4*D*	rTgFKBP5 (*N* = 10), WT (*N* = 10), tTA (*N* = 10)	M and F	4–6 months old	One-way ANOVA with Tukey *post hoc* test	Quadrant time	rTgFKBP5: *F* = 2.038; WT: *F* = 15.22; tTA: *F* = 31.26	rTgFKBP5: *p* = 0.1259; WT: *p* < 0.0001; tTA: *p* < 0.0001
5*A*	rTgFKBP5 (*N* = 5; *n* = 18), WT (*N* = 6; *n* = 29), tTA (*N* = 5; *n* = 22)	M and F	2–3.5 months old	Two-way repeated-measures ANOVA with Bonferroni *post hoc* test	Genotype × time	*F* = 4.820; DFn = 158	*p* < 0.0001
					Genotype	*F* = 1036 DFn = 2	*p* < 0.0001
					Time	*F* = 103.1; DFn = 79	*p* < 0.0001
				One-way ANOVA with Tukey *post hoc* test	Genotype	*F* = 872.5	*p* < 0.0001
5*B*	rTgFKBP5 (*N* = 7; *n* = 16), WT (*N* = 6; *n* = 23), and tTA (*N* = 8; *n* = 26)	M and F	2–3.5 months old	Two-way repeated-measures ANOVA with Bonferroni *post hoc* test	Genotype × time	*F* = 2.484; DFn= 164	*p* < 0.0001
					Genotype	*F* = 344.9; DFn = 2	*p* < 0.0001
					Time	*F* = 117.6; DFn = 82	*p* < 0.0001
				One-way ANOVA with Tukey *post hoc* test of last 5 min of LTP	Genotype	*F* = 154.1	*p* < 0.0001
6*A*	rTgFKBP5 (*N* = 15; *n* = 65), WT (*N* = 15; *n* = 62), and tTA (*N* = 19; *n* = 77)	M and F	2–3.5 months old	Two-way repeated-measures ANOVA with Bonferroni *post hoc* test	Genotype × time	*F* = 2.562; DFn = 58	*p* < 0.0001
					Genotype	*F* = 158.60; DFn = 2	*p* < 0.0001
					Time	*F* = 515.25; DFn = 29	*p* < 0.0001
6*B*	rTgFKBP5 (*N* = 15; *n* = 65), WT (*N* = 15; *n* = 62), and tTA (*N* = 19; *n* = 77)	M and F	2–3.5 months old	Two-way repeated-measures ANOVA with Bonferroni *post hoc* test	Genotype × time	*F* = 1.763; DFn = 58	*p* = 0.0004
					Genotype	*F* = 117.2; DFn = 2	*p* < 0.0001
					Time	*F* = 239.1; DFn = 29	*p* < 0.0001
6*D*	rTgFKBP5 (*N* = 31; *n* = 175), WT (*N* = 33; *n* = 198), and tTA (*N* = 30; *n* = 181)	M and F	2–3.5 months old	Two-way repeated-measures ANOVA with Bonferroni *post hoc* test	Genotype × time	*F* = 0.7413; DFn = 28	*p* = 0.8337
					Genotype	*F* = 44.78; DFn = 2	*p* < 0.0001
					Time	*F* = 50.97; DFn = 14	*p* < 0.0001
7	rTgFKBP5 (*N* = 9; *n* = 21), WT (*N* = 9; *n* = 23), and tTA (*N* = 10; *n* = 31)	M and F	2–3.5 months old	Two-way repeated-measures ANOVA with Bonferroni *post hoc* test	Genotype × time	*F* = 2.557 DFn = 158	*p* < 0.0001
					Genotype	*F* = 561.9; DFn = 2	*p* < 0.0001
					Time	*F* = 33.52; DFn = 79	*p* < 0.0001
				One-way ANOVA with Tukey *post hoc* test of last 5 min of LTD	Genotype	*F* = 2065	*p* < 0.0001
8*B*	rTgFKBP5 (*N* = 4; *n* = 8), WT (*N* = 2; *n* = 4) and tTA (*N* = 2; *n* = 4)	M and F	2–3 months old	Mann–Whitney test exact significance (two-tailed)	Between genotypes at 2 min	*p* = 0.3429	
					Between genotypes at 5 min	*p* = 0.0286	
					Between genotypes at 10 min	*p* = 0.0286	
					Between genotypes at 30 min	*p* = 0.1143	

## Results

### Generation of the rTgFKBP5 mice and the characterization of *FKBP5* expression

In an effort to understand the role of FKBP5 in learning and memory (Binder et al., 2008; Tyrka et al., 2015), we generated novel transgenic mice to overexpress FKBP51 throughout the forebrain. These mice were generated by crossing mice expressing the tetracycline (Tet)-Off transactivator (tTA) driven by a CaMKIIα promoter (CaMKIIα-tTA) with our novel transgenic mice expressing human *FKBP5* under control of a Tet-responsive element, yielding bigenic offspring which included: WT, tTA, FKBP5, and mice positive for both tTA and FKBP5, deemed rTgFKBP5. It has previously been shown that this bigenic strategy leads to robust forebrain expression, which starts at approximately one week postnatal and progressively increases to adult levels by three weeks postnatal due to the activity of the CaMKIIα promoter (Kelly and Vernon, 1985; Mayford et al., 1996; Wei et al., 2004; for details of transgenic construction and validation, see Fig. 1*A–C*). Quantitative real-time PCR revealed that human *FKBP5* expression was ∼12-fold higher in adult rTgFKBP5 mice relative to mouse *Fkbp5* mRNA in WT littermates (Fig. 2*A*). Mouse *Fkbp5* levels were unaffected by the exogenous transgene, suggesting an absence of feedback regulation. Western blot analysis of hippocampal lysates from rTgFKBP5 mice revealed dramatically higher levels of FKBP51 protein compared to WT, FKBP5 single, and tTA littermates (Fig. 2*B*). In fact, 50 µg of protein from the hippocampus of WT, tTA, and FKBP5-singles was similarly immunoreactive to anti-FKBP51 antibody as 10 µg of protein from the bigenic mice (Fig. 2*C*). Elevated human FKBP51 was observed throughout the corticolimbic system including the hippocampus, striatum, and cortices by both immunohistochemistry (Fig. 2*D*,*E*) and Western blotting (Fig. 2*F*,*G*), consistent with the expression pattern of the CaMKIIα-tTa (Wang et al., 2013). To understand the effects of this FKBP51 overexpression, we analyzed the rTgFKBP5 mouse model for general phenotypes, motor ability, and depression-like behavior as well as learning and memory compared to both the WT and tTA control littermates.

### rTgFKBP5 mice have normal body weight, motor function, and unchanged depression-like and anxiety-like phenotypes but display reduced pleasure-seeking behavior

The overexpression of FKBP51 in mice did not lead to any alteration in gross body weight in rTgFKBP5 mice compared to littermate controls (Fig. 3*A*). rTgFKBP5 mice also displayed no significant change in rotarod ability or total distance traveled in the open field test compared to controls, indicating FKBP51 did not alter locomotor function (Fig. 3*B*,*C*). rTgFKBP5 mice also spent similar time in the center zone compared to controls (Fig. 3*D*), suggesting general anxiety was unaffected (Bailey and Crawley, 2009). Since mice lacking FKBP51 have been shown to be protected from depression-like behaviors (O'Leary et al., 2011; Touma et al., 2011), we investigated whether the inverse was true in mice overexpressing FKBP51. Contrary to what we expected, we found that immobility times in the Porsolt FST and TST were unchanged from littermate controls (Fig. 3*E*,*F*). However, when exposed to the reward-based sucrose presence test, rTgFKBP5 mice consumed less sucrose-supplemented water than the tTA control counterparts in the reward-based sucrose preference test (Fig. 3*G*), suggesting increased anhedonia (Strekalova et al., 2011). In fact, they showed equal preference to the water-only and sucrose containing bottles. Interestingly, humans suffering from depression display decreased reward-seeking behavior that can be rescued by antidepressants (Wichers et al., 2009). Taken together, these findings demonstrate that corticolimbic *FKBP5* expression does not affect gross body weight or motor function, while having a modest impact HPA axis function, feedback and associated phenotypes, at least in the absence of environmental factors such as stress.

### rTgFKBP5 mice have slowed spatial reversal learning and impaired cognitive flexibility

**Figure 4.**
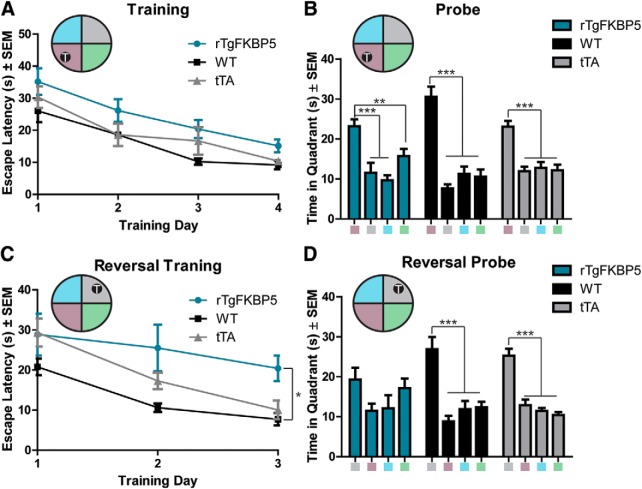
rTgFKBP5 exhibit reversal deficits MWM reversal learning and memory. ***A***, Escape latencies (s) ± SEM from rTgFKBP5, WT, and tTA control mice (*N* = 10/genotype) over 4 d of training in the MWM behavioral task. Two-way ANOVA of entire training. The probe trial ± SEM from (***B***) rTgFKBP5, WT, and tTA mice 24 h after the last training session; ***p < 0.001, **p < 0.01 by one-way ANOVA. ***C***, Escape latencies (s) ± SEM of rTgFKBP5, WT, and tTA mice (*N* = 10/genotype) trained to find an escape platform in the opposite quadrant of the initial MWM; ***p < 0.001 by two-way ANOVA. ***D***, The reversal probe trial ± SEM from rTgFKBP5, WT, and tTA mice; ***p < 0.001 by one-way ANOVA.

Next, we sought to determine the consequences of increased FKBP51 levels on cognitive function. Using the MWM, which is a spatial learning and memory task that has been shown to be hippocampal-dependent, we found that rTgFKBP5 mice trained to the location of the escape platform similarly to their control (WT and tTA) littermates (Fig. 4*A*), and all genotypes of mice were able to recall the location quadrant of the escape platform in the probe trial (Fig. 4*B*). However, when the location of the escape platform was moved to the reversed quadrant, rTgFKBP5 mice had significantly impaired escape latency during the training days (Fig. 4*C*), suggesting a deficit in re-training and re-acquisition, and, when tested by the reversal probe trial, rTgFKBP5 mice were unable to identify the target quadrant significantly from any other quadrant (Fig. 4*D*). This was unlike both control groups, which showed significant preference for the new target quadrant, as expected. Together, these data suggest that FKBP51 overexpression impaired spatial inhibitory learning.

### Overexpression of *FKBP5* modestly enhances hippocampal LTP

**Figure 5.**
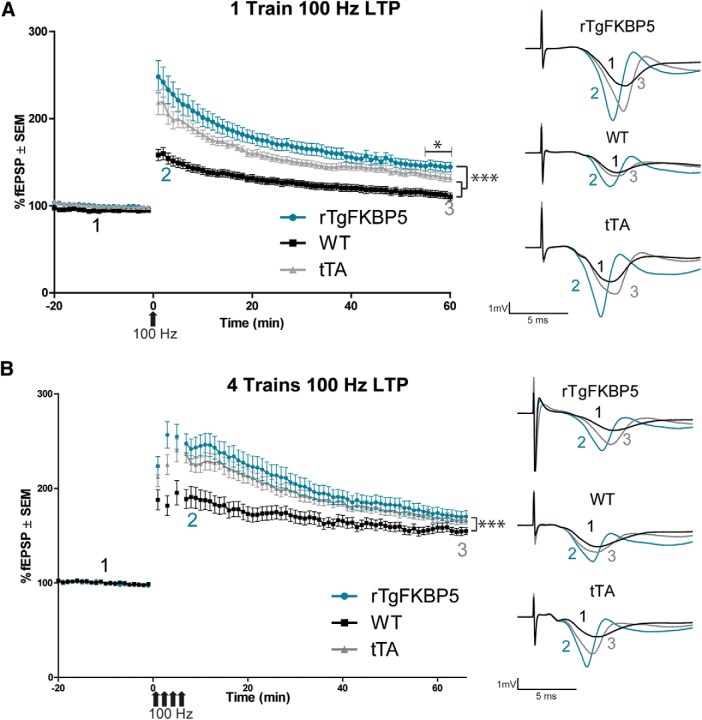
FKBP51 overexpression modestly alters LTP induction and maintenance. *Ex vivo* hippocampal slices from rTgFKBP5 and littermate controls were prepared for LTP testing in the Schaffer collateral pathway. Following a 20-min baseline, slices were stimulated by one-train (***A***) or four-trains (***B***) of HFS at 100 Hz to induce LTP. Evoked fEPSPs ± SEM, normalized to baseline, were measured for 60 min following HFS. In the one-train protocol, slices from rTgFKBP5 (*N* = 5), WT (*N* = 6), and tTA (*N* = 5) mice were used. In the four-train protocol, rTgFKBP5 (*N* = 7), WT (*N* = 6), and tTA (*N* = 8) mice were used. Representative traces are shown: 1 (black) indicates baseline, 2 (teal) indicates initial early LTP potentiation in the first 3 min following potentiation, and 3 (gray) indicates late LTP in the last 3 min of recording. *p < 0.0001 by one-way ANOVA with Tukey’s Multiple Comparison’s test of the last 5 minutes. ***p < 0.0001 by two-way ANOVA.

**Figure 6.**
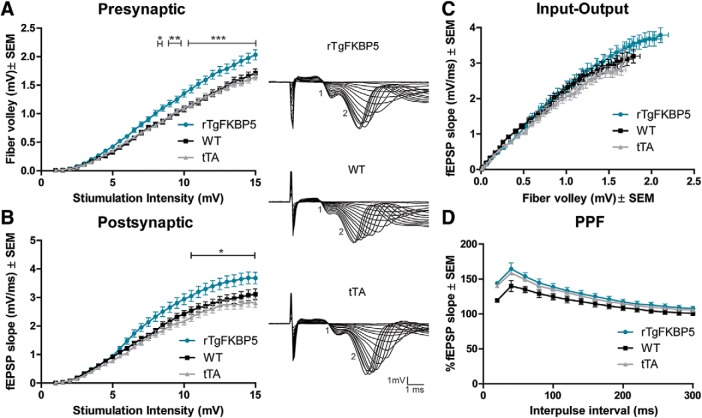
FKBP51 overexpression alters basal AMPA receptor signaling. Following 10 min of consistent response to voltage stimulus, threshold voltage for evoking a fEPSP was measured. Voltage was progressively increased at a rate of 0.5 mV until the maximum fEPSP was reached. The absolute I-O curves of the (***A***) presynaptic fiber volley amplitude (mV) ± SEM and (***B***) postsynaptic fEPSP slope (mV/ms) ± SEM from rTgFKBP5 (*N* = 15), WT (*N* = 15), and tTA (*N* = 19) mice. Significance was determined by two-way ANOVA. ***C***, The I-O curves of the fEPSP slope (mV/ms) ± SEM versus the fiber volley amplitude (mV) ± SEM. Representative traces of the I-O for each genotype are shown with 1 representing fiber volley and 2 representing the fEPSP. ***D***, The PPF from rTgFKBP5 (*N* = 31), WT (*N* = 33), and tTA (*N* = 30) is shown, which is derived from the ratio of the slope of the first peak to the slope of the second peak, over the interstimulus interval (ms). Significance was determined by two-way ANOVA. *p < 0.05, **p < 0.01, ***p < 0.0001 by two-way ANOVA.

Hippocampal LTP is a well-known cellular correlate of memory formation (Luscher and Malenka, 2012). We used a HFS protocol to induce LTP in this model. Using a single 100-Hz stimulation, LTP was modestly, but significantly, enhanced and maintained in hippocampal slice cultures from rTgFKBP5 mice compared to both WT and tTA control animals (Fig. 5*A*), suggesting an increase in synaptic strength in rTgFKBP5 mice through AMPA receptor modulation (Dong et al., 2013). While enhanced LTP is also a phenotype of the tTA mice (Han et al., 2012), the maintenance of LTP in rTgFKBP5 mice was still significantly greater than tTA mice. When we exposed slices in a parallel experiment to four trains of 100-Hz stimulations, we found that the modest LTP enhancement over the tTA controls was no longer detected using this stimulation protocol (Fig. 5*B*). To determine the contribution of the presynapse and postsynapse, we analyzed the ratio of the input stimulus to evoke output activity. This I-O revealed that both the fiber volley and the slope of the EPSP (fEPSP) were significantly higher following the same stimulation intensity in rTgFKBP5 mice compared to littermate controls (Fig. 6*A–C*). This suggests that FKBP51 may be altering both pre- and post- synaptic signaling. PPF was unchanged in rTgFKBP5 mice from controls (Fig. 6*D*), suggesting that these effects are not due to calcium-mediated differences or short-term synaptic plasticity (Zucker and Regehr, 2002; Han et al., 2012). Collectively, these electrophysiology data suggest that hippocampal FKBP51 increases can promote excitability of neurons, which implicates a role in the regulation of AMPA receptors.

### Overexpression of *FKBP5* impairs hippocampal long-term depression (LTD) by accelerating AMPA receptor recycling

**Figure 7.**
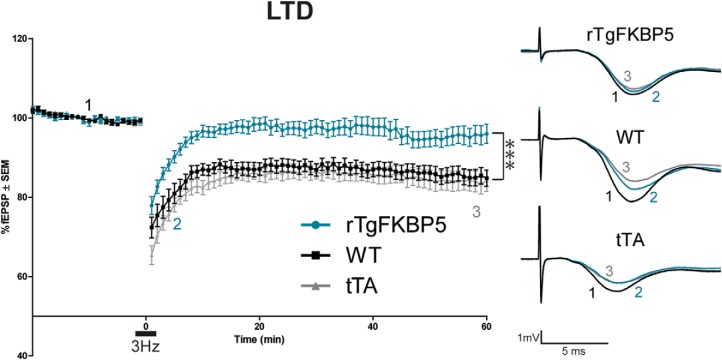
LTD is impaired in rTgFKBP5 mice. LTD fEPSP (%) ± SEM was induced following 20-min baseline recording in *ex vivo* slices from rTgFKBP5 (*N* = 9), WT (*N* = 9), and tTA (*N* = 10) mice by 900 pulses of low-frequency (3 Hz) stimulation. Representative traces are shown: 1 (black) indicates baseline, 2 (teal) indicates early LTD, and 3 (gray) indicates late LTD; ***p < 0.001 determined by two-way ANOVA.

To further examine the possibility of AMPA receptor regulation by FKBP51, LFS-induced LTD was performed. Compared to control littermates, rTgFKBP5 mice displayed significantly impaired LTD (Fig. 7), in fact, their slopes returned to baseline levels around 10 min into the recording. Together, this provides additional evidence that AMPA receptor activation or localization to the synaptic membrane was altered in mice overexpressing FKBP51 (Dong et al., 2013).

**Figure 8.**
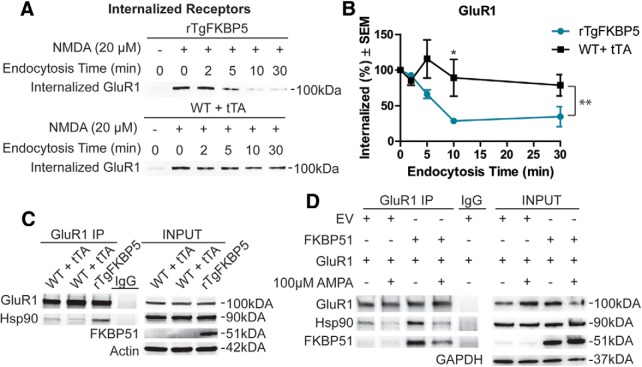
FKBP51/Hsp90 bind to GluR1-type AMPA receptors to regulate trafficking. ***A***, Representative Western blottings form biotinylation assays of receptor endocytosis was performed on *ex vivo* slices, as described in Materials and Methods, rTgFKBP5 (*N* = 4; *n* = 8), WT (*N* = 2; *n* = 8), and tTA (*N* = 2; *n* = 8). Following labeling with Sulfo-NHS-SS biotin and chemical LTD (20 µM NMDA; 5 min) treatment, receptors were permitted to externalize at 30°C for the indicated times. ***B***, The quantification ± SEM of multiple acquisitions is shown for GluR1. ***C***, Representative Western blottings from anti-GluR1 co-immunoprecipitations and corresponding inputs from control and rTgFKBP5 mice immunoblotted as indicated. rTgFKBP5 (*N* = 2), WT (*N* = 2), and tTA (*N* = 2) total from two independent experiments. ***D***, Representative Western blottings of anti-GluR1 co-immunoprecipitations and corresponding inputs from HEK293T cells transfected with GluR1 and FKBP51 or empty vector (EV) for 48 h were immunoblotted with antibodies as indicated. Just before harvest, cells were treated with 100 µM AMPA or PBS for 10 min to induce GluR1 receptor internalization. *p = 0.0286 by t-test of this time point. **p < 0.001 by two-way ANOVA.

Since GluR1-type AMPA receptor endocytosis has been correlated with LTD (Lee et al., 2010), we next examined whether GluR1 receptor recycling was altered in the hippocampus of rTgFKBP5 mice. Once internalized these receptors can have two fates, they can be degraded by macroautophagy or be recycled back to the membrane surface (Ehlers, 2000). Using *ex vivo* hippocampal slices from rTgFKBP5 and control mice, we biotinylated all of the surface receptors and then stimulated internalization by a chemical LTD protocol (Lee et al., 1998). Following LTD stimulation, slices were incubated for up to 30 min, as indicated. Once the designated time has passed extracellular biotin was cleaved and the internalized receptors were analyzed by immunoprecipitation (Fig. 8*A*). Quantification of the internalized receptors (Fig. 8*B*) revealed marked differences in the maintenance of internalized GluR1 receptors in rTgFKBP5 mice compared to littermates following chemically-induced LTD. In line with our electrophysiology results, rTgFKBP5 mice were unable to maintain the internalized GluR1 receptor pool as well as their control littermates. These assays were performed in the presence of leupeptin, a macroautophagy inhibitor, to preserve the internalized receptors that are not fated for recycling back to the surface. Thus, since rTgFKBP5 mice had a rapid loss of internalized GluR1 receptors compared to control mice, these findings indicate that FKBP51 promotes recycling of AMPA receptors back to the membrane.

### FKBP51 enhances GluR1-type AMPA receptor binding to Hsp90

It was previously shown that Hsp90 can regulate AMPA receptor cycling through an interaction with the tetratricopeptide repeat (TPR) domain (Gerges et al., 2004). Therefore, we speculated that since FKBP51 contains a TPR domain and is a known Hsp90 co-chaperone, it was regulating GluR1-type AMPA receptor trafficking through its association with Hsp90. Indeed, more Hsp90/GluR1 complexes were present in hippocampal lysates from rTgFKBP5 mice compared to littermates (Fig. 8*C*). We corroborated these results in a HEK293T cell model, which was transfected with GluR1 and FKBP51 as indicated. Co-immunoprecipitation revealed that GluR1 can interact with both FKBP51 and Hsp90 (Fig. 8*D*) with and without AMPA treatment to stimulate GluR1 internalization (Ehlers, 2000). Moreover, increased FKBP51 levels enhanced the association of Hsp90 with GluR1, suggesting the high levels of FKBP51 can stimulate AMPA receptor recycling by increasing the interaction between Hsp90 and GluR1 impacting synaptic plasticity.

## Discussion

**Figure 9.**
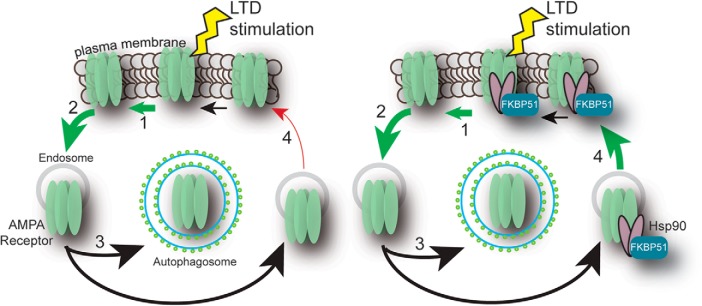
Working schematic of FKBP51/Hsp90 regulation of AMPA receptor recycling. Upon LTD stimulation, AMPA receptors are (1) moved away from the synapse and (2) internalized, following internalization either degraded through (3) macroautophagy or (4) recycled back to the membrane. LTD is expressed when the rate of internalization is greater than the rate of exocytosis. In the presence of high levels of FKBP51, Hsp90 binds to GluR1 and drives more AMPA receptors to be recycled back to the membrane.

*FKBP5* is a risk factor in a growing number of affective disorders with diverse symptoms, particularly those arising from childhood trauma (Zannas et al., 2016). Our results identify a previously unknown function for FKBP51 in inhibitory learning and bidirectional synaptic plasticity that could underlie some of the phenotypes observed in patients. In this way, the rTgFKBP5 mouse model could prove a valuable tool for preclinical investigation of therapies for this group of disorders. Using this transgenic model, we have found that FKBP51 contributes to the fate decisions of internalized GluR1-type AMPA receptors by increasing the association with Hsp90. This interaction specifically accelerates the recycling rate of GluR1 receptors to the synaptic membrane during synaptic activity (Fig. 9). This is the first time that FKBP51 has been shown to regulate GluR1-type AMPA receptors and could explain how elevations in *FKBP5* relate to diverse phenotypes in psychiatric disorders and other diseases.

This work highlights a novel way that FKBP51 can regulate synaptic plasticity and membrane receptor trafficking. We found that spatial reversal learning was significantly impaired in rTgFKBP5 mice as was LTD, which has been shown to be a mediator of reversal learning (Dong et al., 2013). At the same time, rTgFKBP5 mice had an enhancement of LTP and an increase in neuronal excitability, but no cognitive improvements were measured. It is possible that the age difference in the mice used for behavior (four to six months old) compared to the mice used for electrophysiology (2–3.5 months old) could have an impact on the dynamics of learning and memory, although we do not expect there to be significant differences in learning and memory over this timeframe, it is possible. However, previous studies have demonstrated that LTP can be enhanced independently of performance on cognitive tasks (Schmidt et al., 2011). Interestingly, it has also been demonstrated that LTP can inhibit LTD, and that this is through the glycogen synthase kinase (GSK)-3β and AKT (also known as protein kinase B) pathways (Peineau et al., 2007), a pathway that FKBP51 has been shown to directly regulate through pleckstrin homology domain leucine-rich repeat protein phosphatase (PHLPP; Wang, 2011; Gassen et al., 2014). Potentially FKBP51 is altering LTD and learning and memory through regulation of this pathway. In addition, FKBP51 can increase autophagic activity (Gassen et al., 2015) and, thus, could regulate synaptic plasticity by altering the turnover of many components including AMPA receptors. Additional studies are needed to better understand the crosstalk between FKBP51 and synaptic plasticity.

Our findings also highlight a novel role for *FKBP5* in age-related cognitive decline and psychiatric illness, since *FKBP5* levels progressively increase in these disorders (Blair et al., 2013; Sabbagh et al., 2014; Zheng et al., 2016). Reversal learning and cognitive flexibility are negatively impacted by aging (Glisky, 2007; Gajewski et al., 2010; Schoenfeld et al., 2014), a phenotype that was observed in the rTgFKBP5 mouse model (Fig. 4*A–D*). Moreover, LTD impairments have been reported in aged animals (Lee et al., 2005; Potier et al., 2010), consistent with our model (Fig. 7). Perhaps FKBP51 contributes to PTSD symptoms by disrupting the ability to extinguish memories, therefore allowing individuals to better maintain intense memories of a traumatic event. While future studies are still needed to better understand these connections, our findings suggest that FKBP51 could contribute to a number of age-related cognitive pathologies, possibly partly through a role in regulating AMPA receptor recycling and synaptic plasticity.

Unlike FKBP51, Hsp90 has been previously revealed to be necessary for synaptic protein recycling and neurotransmitter release (Gerges et al., 2004; Chen et al., 2014), our results show that levels of Hsp90 co-chaperones can influence receptor fate as well. It is likely that Hsp90 coordinates directly with FKBP51 as well as other TPR containing co-chaperones, like FKBP52 and cyclophilin 40, to interact with glutamate receptors and influence synaptic function. In addition, Hsp90 has been shown to coordinate with the heat shock cognate (Hsc) 70/cysteine string protein α (CSPα) complex at the synaptic membrane to regulate clathrin-dependent endocytosis of glutamate receptors (Jiménez et al., 2002; Sakisaka et al., 2002; Cottrell et al., 2004).

Chaperone-mediated synaptic trafficking has also been implicated in neurodegenerative disease (Gerges et al., 2004; Köroğlu et al., 2013; Chen et al., 2014; Vilariño-Güell et al., 2014). For example, mutations in the Hsp70 co-chaperones DNAJC13 and DNAJC6 that reduce clathrin-mediated endocytosis cause Parkinson’s disease (Köroğlu et al., 2013; Vilariño-Güell et al., 2014). Also, the endoplasmic reticulum (ER) resident Hsp90, glucose-regulated protein 94 (Grp94), impairs the trafficking of a mutant, aggregation-prone protein to the lysosome, causing it to accumulate and promote hereditary glaucoma (Suntharalingam et al., 2012). More generally, chaperone dysfunction has been linked to the trafficking of proteins between distinct organelles in disease. For example, despite still being functional, mutant cystic fibrosis transmembrane conductance regulator (CFTR) is trafficked out of the ER for degradation by Hsp90 when in complex with its co-chaperone Aha1 resulting in disease (Wang et al., 2006). Now, in light of our findings, TPR-containing chaperones also play a critical role in chaperone-mediated synaptic trafficking. In fact, in a recent study in postmortem human brains, Young et al. (2015) suggested that FKBP51 may regulate neuronal spine densities. It is possible that the increased levels of FKBP51 measured by that study impaired spine morphogenesis directly altered AMPA receptor recycling, since spine size and density can be directly regulated by AMPA receptor trafficking (Matsuzaki et al., 2004). Thus, the function of chaperones appears to extend beyond regulation of protein folding to vital and dynamic synaptic trafficking mechanisms.

Efforts to better understand the AMPA receptor cycle have recently been under intense investigation. Internalized AMPA receptors are trafficked to the early endosome where they are either transferred to the lysosome for degradation or to the recycling endosomes (REs) as a replenishment pool for membrane reinsertion (Ehlers, 2000). But our understanding of the mechanism contributing to these processes remains limited. It is known that posttranslational modifications, such as palmitoylation, ubiquitination, and most notably phosphorylation, regulate AMPA receptor trafficking. AMPA receptor trafficking is also regulated by interactions with a variety of AMPA receptor interacting proteins (Anggono and Huganir, 2012; Lu and Roche, 2012). Stability of AMPA receptors within the synaptic membrane is supported by a number of these interacting proteins including: postsynaptic density (PSD)-95, stargazin, glutamate receptor-interacting protein (GRIP)1, GRIP2, and synaptic associated protein (SAP)97. AMPA receptor endocytosis is promoted by PICK1 (Lu and Roche, 2012), which coordinates with protein kinase C and casein kinase II substrate in neurons (PACSIN) during synaptic activity to regulate the internalization and recycling of AMPA receptors (Widagdo et al., 2016). Transferrin receptor (TFR) also promotes endocytosis of AMPA receptors in synaptically active neurons (Liu et al., 2016). Until recently, less has been known about the recycling arm of AMPA receptor trafficking. N-ethymaleimide-sensitive factor (NSF; Huganir and Nicoll, 2013), the µ3A subunit of AP-3 (Steinmetz et al., 2016), and KIBRA (Heitz et al., 2016) all can promote recycling of AMPA receptors. µ3A, KIBRA and now FKBP51 have each been shown to specifically regulate recycling of GluR1-type AMPA receptors following activity dependent internalization. Thus, our findings identify a novel regulator of the decision-making process governing GluR1-type AMPA receptor trafficking in active synapses.

We were surprised that FKBP51 overexpression did not lead to more phenotypes related to depression-like and anxiety-like behaviors. We hypothesize these phenotypes may be precipitated in the rTgFKBP5 model in the presences of environmental factors, such as stress. It is also possible that we did see these phenotypes due to limitations in our current study, such as using both male and female mice throughout, the location of FKBP51 expression, or the age of the mice. Follow-up studies are needed in this model using different Tet driving promoters as well as careful investigation in to the combinatory effects of environmental factors, including stress, as well as sex-related differences to better illuminate how alterations in FKBP51 may increase susceptibility of PTSD, depression, and other psychiatric disorders.

In conclusion, our results support a model in which FKBP51 promotes the regulation of GluR1-type AMPA receptor trafficking through Hsp90 leading to altered neurotransmission and reduced cognitive flexibility in rTgFKBP5 mice. In this way, disease-associated SNPs and stressors that increase *FKBP5* expression can influence the brain in an unexpected way. Not only can FKBP51 regulate GR activity (Rein, 2016), but it may also have a major impact on glutamatergic neurotransmission and synaptic plasticity.

## Synthesis

Reviewing Editor: Jeffrey Blaustein, University of Massachusetts Decisions are customarily a result of the Reviewing Editor and the peer reviewers coming together and discussing their recommendations until a consensus is reached. When revisions are invited, a fact-based synthesis statement explaining their decision and outlining what is needed to prepare a revision will be listed below. The following reviewer(s) agreed to reveal their identity: Mathias Schmidt.

18 Please comment on whether this captures everything. Thanks!

There are several major issues that need to be addressed in revision. The most important are as follows. More detail and some minor issues are included in the specific reviews that follow:

1. The antibody must be characterized more fully, as it is important to assess the meaning of the data.

2. The statistical analysis needs more work as discussed below.

3. Please discuss the seeming discrepancy of decreased cognition in the face of increased LTP.

4. Provide the age of the mice throughout.

5. Include sex/gender as a co-factor in the analyses. If this is not possible, at least discuss the fact that both males and females were used.

6. Discuss in greater detail the already documented effects of FKP51 to put these results in perspective.

Reviewer 1

Overall, the study addresses a highly relevant question and contributes interesting new insight into FKBP51 function. It is recommended to address the following points:

Abstract: please change “...., which transcribes the FKBP51 protein” . Further down, it is stated that FKBP51 works with Hsp90. While this is quite plausible, the study does not provide actual proof for it. The fact that the receptor IP brings down both FKBP51 and Hsp90 does not necessarily mean that they are in the same complex.

Introduction, second sentence: The SNPs the authors refer to are associated with higher expression and with the disease parameters the authors describe (Binder et al. 2004 actually is a study on depression, not PTSD). This may or may not mean that higher FKBP51 protein expression facilitates the precipitation of these diseases. The studies have not shown this (could also be the altered stress reactivity of the gene, e.g.).

Figure 2, comparison of the protein levels of the endogenously produced FKBP51 with the levels from the transgene. To make this comparison valid, the antibody used needs to be characterized better. The field is plagued by the lack of antibodies that efficiently recognize mouse FKBP51. Available antibodies typically produce a strong and specific signal with human FKBP51, but not with mouse FKBP51. Therefore, validation assays for the antibody are required to make the presented comparison meaningful. The RNA data already suggest a strong overexpression of the human FKBP51 (sufficient to make the case), but whether on top of this the translation efficiency also is much stronger is questionable.

Page 14, line 293, “....the consequence of this mechanism....”: which mechanism? The overexpression?

Page 16, line 342: it appears useful to mention the studies linking FKBP51 to autophagy (there or in the introduction) to better understand why an autophagy inhibitor (inhibitor of lysosomal proteases) is used.

Figure 7C, why was FKBP51 not probed for in the IP?

Discussion of LTP and memory: In general, improved memory is associated with increased LTP (e.g. J Neurosci 2007, vol27:10487-10496). Therefore, it would help discussing the observation of enhanced FKBP51 causing increased LTP and decreased memory function. Thus, it would be worth mentioning examples where LTP changes in brain slice preparations do not correlate with learning and memory in the "usual" way, for example Prog. Neuropsychopharmacol Biol Psychiatry 1997, vol 21:47-68. Nat Neurosci 2002, vol 5:868-873. PNAS 2011, vol. 108:17213-17218

Reviewer 2

Tthe data are an important addition to the existing literature on this psychiatric risk factor, but there are several concerns that the authors should address in a revised version of their manuscript:

a. The authors present a new mouse model, but the characterization of this model is only performed regarding cognition. What about other physiological/behavioral parameters. I would expect at least data on weight, development, appearance etc. Given the close link of FKBP51 to HPA axis function, at least some basic measures (e.g. morning/evening corticosterone, adrenal weight, etc.) should be provided, as alterations in HPA axis function may also impact on cognitive flexibility and AMPA receptor signaling. Further, the animals should be phenotyped on general anxiety and locomotion, to exclude any confounding effects on MWM behavior. (For the purposes of this revision, it is only necessary to discuss the other effects of FKBP51 as discussed above.)

b. Throughout their studies the authors used mixed sex groups including both males and females. This is unusual, given the differential behavior of male and female mice, their differential physiology (including e.g. a different HPA axis function), etc. In addition, the estrus cycle of female mice affects both behavior and physiology, and should be taken into account. If possible, the authors should include gender as a co-factor in their analyses and mention this caveat as limitation in the discussion.

c. The age of the mice at testing (e.g. for the MWM) was not always stated. Was the age of the mice the same as for the electrophysiological recordings (2 months)? This information is critical, given the age-dependent decline of cognition.

d. Please always indicate how many animals per group were used (missing e.g. for the co-IPs)

e. The statistical analysis and reporting of the data needs to be drastically improved. Please report F and p values for all ANOVA tests and indicate in the case of 2-way ANOVAs the main effects as well as interaction effects. In case of a significance, post-hoc tests should be performed (indicate the type of post-hoc test used) and their p value needs to be reported as well. Further, please indicate if the data were tested for normal distribution - if not (or due to too few animals per group not applicable) please utilize non-parametric tests.

f. Why is the native FKBP51 expression so low / not detectable in the immunohistochemistry and western blot analyses? Can the authors confirm that the antibody indeed detects also the native mouse FKBP51 protein and is indeed specific for FKBP51?

g. For the immunohistochemistry it would be nice to include an overview picture of a representative section and also schematic drawings of the region of interest.

i. The observed increase in LTP would potentially suggest an improved cognition, which the authors did not observe. This should be extensively discussed.

## Author Response

Synthesis Statement for Author (Required):

There are several major issues that need to be addressed in revision. The most important are as follows. More detail and some minor issues are included in the specific reviews that follow:

1. The antibody must be characterized more fully, as it is important to assess the meaning of the data.

2. The statistical analysis needs more work as discussed below.

3. Please discuss the seeming discrepancy of decreased cognition in the face of increased LTP.

4. Provide the age of the mice throughout.

5. Include sex/gender as a co-factor in the analyses. If this is not possible, at least discuss the fact that both males and females were used.

6. Discuss in greater detail the already documented effects of FKBP51 to put these results in perspective.

Reviewer 1

Overall, the study addresses a highly relevant question and contributes interesting new insight into FKBP51 function. It is recommended to address the following points:

Abstract: please change “...., which transcribes the FKBP51 protein” . Further down, it is stated that FKBP51 works with Hsp90. While this is quite plausible, the study does not provide actual proof for it. The fact that the receptor IP brings down both FKBP51 and Hsp90 does not necessarily mean that they are in the same complex.

Thank you for your comment. Yes, we agree than an IP does not mean they are definitely in the same complex. We have tampered the language at the bottom of the abstract.

Introduction, second sentence: The SNPs the authors refer to are associated with higher expression and with the disease parameters the authors describe (Binder et al. 2004 actually is a study on depression, not PTSD). This may or may not mean that higher FKBP51 protein expression facilitates the precipitation of these diseases. The studies have not shown this (could also be the altered stress reactivity of the gene, e.g.).

Yes, this is true that the cause and effect relationship of high FKBP51 protein has not yet been shown to facilitation these psychiatric diseases. We have reworded this sentence and would like to highlight that that is one of the purposes of conducting the study presented here, to better understand the role and consequences of elevated FKBP51.

Figure 2, comparison of the protein levels of the endogenously produced FKBP51 with the levels from the transgene. To make this comparison valid, the antibody used needs to be characterized better. The field is plagued by the lack of antibodies that efficiently recognize mouse FKBP51. Available antibodies typically produce a strong and specific signal with human FKBP51, but not with mouse FKBP51. Therefore, validation assays for the antibody are required to make the presented comparison meaningful. The RNA data already suggest a strong overexpression of the human FKBP51 (sufficient to make the case), but whether on top of this the translation efficiency also is much stronger is questionable.

Thank you for pointing this out. We know that the Hi51B does react with mouse FKBP51, but we went ahead and tested all the FKBP51 antibodies we had available to see if there were differences in reactivity. In doing this, we have found that Hi51E, an antibody previously described in Gross et all Comp Med 2008, actually reacted best with endogenous mouse FKBP51. We confirmed specificity for mouse FKBP51 by running mouse cell line on a Western with or without Dexamethasone to stimulate FKBP51 expression and {plus minus} some novel GR antagonists that we have been working with in the lab. We have since harvested new tissue and ran Westerns to probe for FKBP51 using this antibody.

Page 14, line 293, “....the consequence of this mechanism....”: which mechanism? The overexpression?

Thank you for pointing out this confusing statement. We have updated the sentence for clarity.

Page 16, line 342: it appears useful to mention the studies linking FKBP51 to autophagy (there or in the introduction) to better understand why an autophagy inhibitor (inhibitor of lysosomal proteases) is used.

We apologize for any confusion. The macroautophagy inhibitor was used to prevent the pool of AMPA receptors that were internalized from being degraded, since it is the major degradation pathway for these receptors and is commonly used in these experiments. And, we agree that the link between FKBP51 and autophagy is interesting and may have some affect AMPA receptor turnover. Whether the FKBP51-mediated autophagy activation alters the turnover of AMPA receptors would be very interesting to investigate, but is outside of this current study. However, since the GluR1receptors in rTgFKBP5 mice had reduced maintenance of intracellular receptors, we expect the main effect of FKBP51 on AMPA receptors to be through recycling rates and not due to activation of autophagy. We have added additional text in the results to clarify the use of the autophagy inhibitor and in the discussion to suggest that FKBP51 could regulate AMPA receptors through autophagy activation.

Figure 7C, Why was FKBP51 not probed for in the IP?

We agree that having FKBP51 in 7C is important, so we probed for this in our samples, however we had a strong heavy chain band at 50kDa in the tissue IP samples, so we were unable to clearly see FKBP51. This was one of the reasons that the experiments presented in 7D were preformed, since we have minimal heavy chain banding from cultured cells.

Discussion of LTP and memory: In general, improved memory is associated with increased LTP (e.g. J Neurosci 2007, vol27:10487-10496). Therefore, it would help discussing the observation of enhanced FKBP51 causing increased LTP and decreased memory function. Thus, it would be worth mentioning examples where LTP changes in brain slice preparations do not correlate with learning and memory in the “usual” way, for example Prog. Neuropsychopharmacol Biol Psychiatry 1997, vol 21:47-68. Nat Neurosci 2002, vol 5:868-873. PNAS 2011, vol. 108:17213-17218

Thank you for your comment. We have added a new paragraph into the discussion to better highlight the enhanced saturation of LTP and ablation of LTD in their relationship with spatial learning. LTP saturation has been previously liked to spatial learning deficits, we have now highlighted this in our discussion as well as added additional possibilities of how to explain this possible disconnect in learning and memory. Additionally, LTD has also been closely correlated with spatial learning and memory, and this was ablated in the rTgFKBP5 mice. Our added discussion, and inclusion of some of the references you suggested here, should hopefully help clarify the electrophysiology and behavioral results.

Reviewer 2

The data are an important addition to the existing literature on this psychiatric risk factor, but there are several concerns that the authors should address in a revised version of their manuscript:

a. The authors present a new mouse model, but the characterization of this model is only performed regarding cognition. What about other physiological/behavioral parameters. I would expect at least data on weight, development, appearance etc. Given the close link of FKBP51 to HPA axis function, at least some basic measures (e.g. morning/evening corticosterone, adrenal weight, etc.) should be provided, as alterations in HPA axis function may also impact on cognitive flexibility and AMPA receptor signaling. Further, the animals should be phenotyped on general anxiety and locomotion, to exclude any confounding effects on MWM behavior. (For the purposes of this revision, it is only necessary to discuss the other effects of FKBP51 as discussed above.)

Thank you for letting us know that this information would be helpful to include. We have now added a new figure (Figure 3) that includes body weight, motor skills, anxiety, depression-like and anhedonia-like behavioral tasks. We hope you will find these additional animal experiments enhanced the findings in this manuscript. We did not include CORT in this paper, but have added discussed it better in the discussion. We performed CORT measurements in these multiple cohorts, since we agree that it is an important readout, and each time we have seen diverse patterns where rTgFKBP5 mice have significantly lower or higher CORT compared to controls, taking into account sex as an independent variable. Because we have had diverse findings, we are now working on a new large cohort with or without stress where we can do a very careful investigation into the CORT dynamics. We hope you will be supportive of us waiting to put this data into our next manuscript, when we are confident in the results. Also, we hope the addition of Figure 3 will satisfy most of your questions about the general well-being of the mice.

b. Throughout their studies the authors used mixed sex groups including both males and females. This is unusual, given the differential behavior of male and female mice, their differential physiology (including e.g. a different HPA axis function), etc. In addition, the estrous cycle of female mice affects both behavior and physiology, and should be taken into account. While it is too late to design the experiment with this in mind, the issue can be commented upon in the discussion. If possible, the authors should include gender as a co-factor in their analyses and mention this caveat as limitation in the discussion.

Yes, we agree that sex is an important variable to investigate in these studies. We have broken apart each of the behavioral tasks by sex, and found that the sex effect in these studies is quite modest. In fact, in most circumstances there is more of a tTA effect than an effect based on FKBP51, with the sole exception being in the Sucrose Preference test. We feel that the inclusion of these graphs in the paper may add confusion rather than clarity. We would predict that in stressed mice, the effects by sex will be more pronounced. We are investigating this in ongoing studies in the lab.

c. The age of the mice at testing (e.g. for the MWM) was not always stated. Was the age of the mice the same as for the electrophysiological recordings (2 months)? This information is critical, given the age-dependent decline of cognition.

We apologize that this was not clearly stated. For clarity, we have now included this information in Table 1, the Summary of statistical analysis. The MWM was performed in mice between 4-6 months of age. The electrophysiology was performed in 2 - 3.5 month mice. Mice for electrophysiology were chosen at that time point due to the stability and reliability of the ex vivo slices for electrophysiology. While we would not expect dramatic cognitive decline over this time difference, we have mentioned this in the discussion as a possibility.

d. Please indicate how many animals per group were used (missing e.g. for the co-IPs) in all cases.

We apologize that this was unclear. This information is now included very clearly in our Summary of statistical analysis in Table 1 along with additional details in Figure 8 legend.

e. The statistical analysis and reporting of the data needs to be drastically improved. Please report F and p values for all ANOVA tests and indicate in the case of 2-way ANOVAs the main effects as well as interaction effects. In case of a significance, post-hoc tests should be performed (indicate the type of post-hoc test used) and their p value needs to be reported as well. Further, please indicate if the data were tested for normal distribution - if not (or due to too few animals per group not applicable), non-parametric tests should be used.

Thank you for pointing this out. We have now tested for Gaussian distribution and used the Mann-Whitney Test where applicable. Our statistical analysis is now summarized in Table 1.

f. Why is the native FKBP51 expression so low / not detectable in the immunohistochemistry and western blot analyses? Can the authors confirm that the antibody indeed detects the native mouse FKBP51 protein and is indeed specific for FKBP51?

We understand your concern. We have now tested a series of FKBP51 antibodies and now are using one that reacts more strongly with mouse FKBP51. For a more detailed explanation and example images confirming specificity, please see our response to reviewer one above.

g. For the immunohistochemistry it would be desirable to include an overview picture of a representative section and also schematic drawings of the region of interest.

Thank you for letting us know that this would be helpful. We have now added a full representative section showing the regions of interest (Figure 2D). We kept this as the Hi51B antibody, because this antibody works best for staining (Figure 2D-E). Therefore, we removed the representative WT mouse images from the figure, since Hi51B reacts less with mouse tau to emphasize the FKBP51 produced form the exogenous transgene.

i. The observed increase in LTP would potentially suggest an improved cognition, which the authors did not observe. This should be discussed.

While we did see in increase in LTP for the 1-train protocol, this effect was no longer detected in the 4-trains, demonstrating that the ceiling for learning overall is not higher, but suggests that it can be achieved by a lower stimulus. LTP saturation has been previously linked to impaired spatial learning in a number of papers including (Barnes et all J Neurosci 1994, Moser Science 1998, Gruart J Neurosci 2006), and may explain why we did not observe improved cognition here . We have now included these details in the discussion and adjusted our wording of “enhanced LTP” to better highlight that we are seeing saturated LTP, since they are not able to increase the total amount of LTP with a higher stimulus. Moreover, acute hippocampal slice learning and MWM may show some differences in learning, because not only the hippocampus is essential for spatial learning, but other brain regions including the amygdala, thalamus, and striatum, and basal forebrain can also impact spatial learning, which are not investigated by LTP. One other possibility, is that it could be possible rTgFKBP5 mice may be displaying signs of increased learning by their lack of ability to unlearn as shown by the deficits in MWM reversal and LTD/DEP, because they are unable to let go of their previous memory.
