# Supplementary figures and images for: The Disease-Associated Chaperone FKBP51 Impairs Cognitive Function by Accelerating AMPA Receptor Recycling
Source: eNeuro. 2019 Mar 1;6(1):ENEURO.0242-18.2019. doi: 10.1523/ENEURO.0242-18.2019 (PMC6450497; doi:10.1523/ENEURO.0242-18.2019)

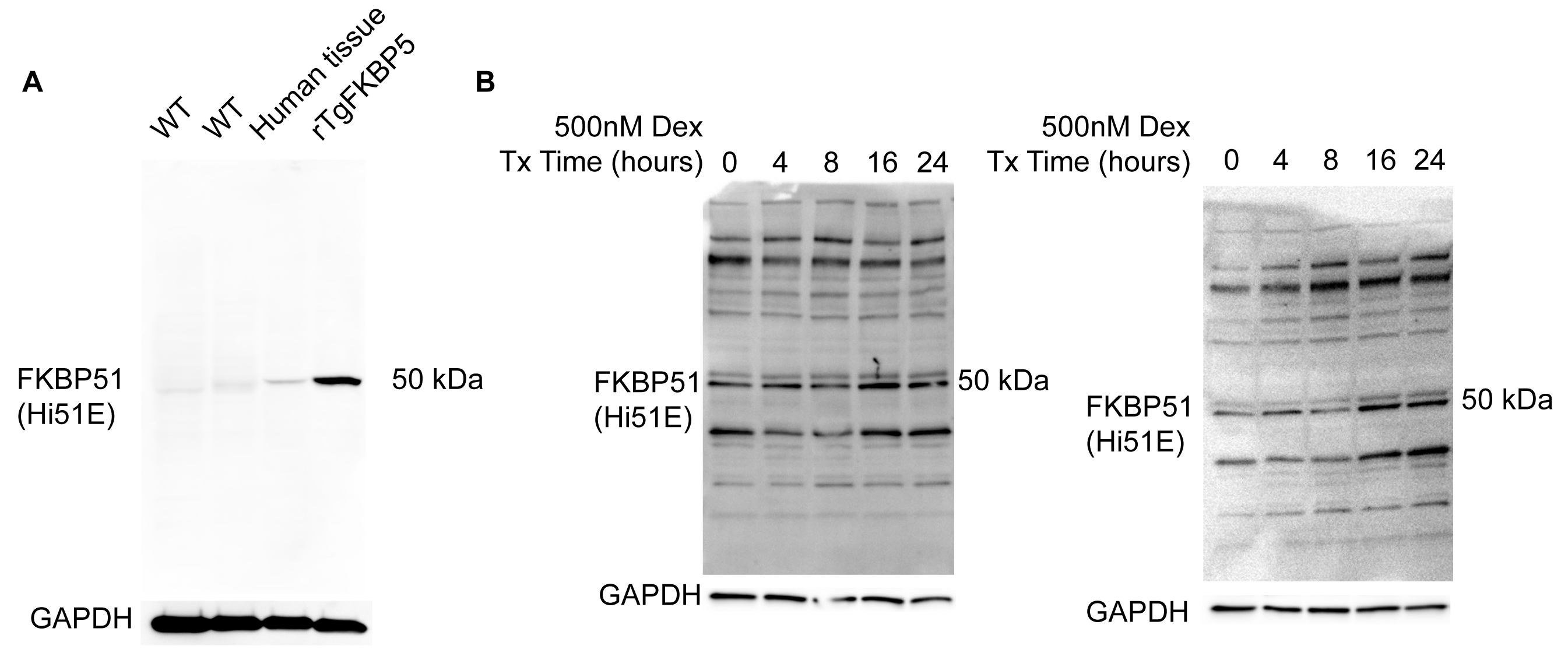

Supplement: Extended Data Figure 2-1 — FKBP51 (Hi51E) antibody recognizes mouse and human FKBP51 in tissue and mouse cell lysates. A, Western blotting of 30 µg of tissue from the hippocampi of two 12-month-old WT mice, tissue from the medial temporal gyrus an 87-year-old male (provided deidentified by the UCI Brain Bank), and tissue from the hippocampus of a one-month-old rTgFKBP5 mouse probed for FKBP51 (Hi51E) and GAPDH antibodies. B, Western blottings from independent experiments using mouse hippocampal HT-22 cells cultured in complete MEM media treated with dexamethasone (Dex) as indicated, to induce FKBP51 expression, probed by FKBP51 (Hi51E) and GAPDH antibodies. Download Figure 2-1, TIF file. [file sup_enu-eN-NWR-0242-18-s02.tif]

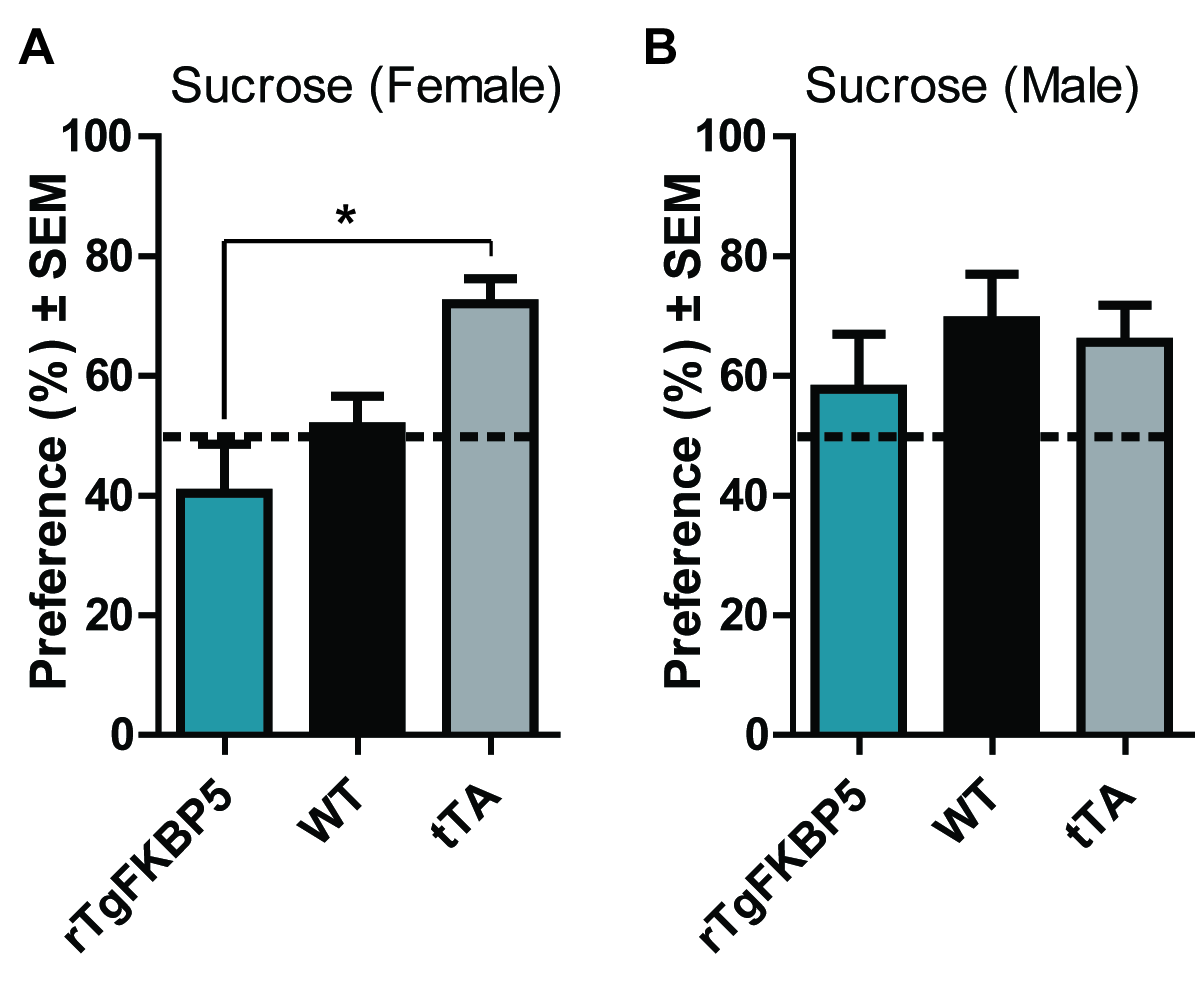

Supplement: Extended Data Figure 3-1 — Anhedonia phenotype in rTgFKBP5 mice is driven by females. Sucrose preference was measured in (A) female rTgFKBP5 (N = 8), WT (N = 10), and tTA (N = 11) and (B) male rTgFKBP5 (N = 8), WT (N = 12), and tTA (N = 9) mice. Consumption was measured by the difference in the weight of bottles filled with sucrose water versus tap water. Sucrose preference percentage ± SEM was determined by the amount (g) of sucrose water consumed versus the amount of total water consumed over the 2-h task. Download Figure 3-1, TIF file. [file sup_enu-eN-NWR-0242-18-s03.tif]
