# Supplementary material for: The Disease-Associated Chaperone FKBP51 Impairs Cognitive Function by Accelerating AMPA Receptor Recycling
Source: eNeuro. 2019 Mar 1;6(1):ENEURO.0242-18.2019. doi: 10.1523/ENEURO.0242-18.2019 (PMC6450497; doi:10.1523/ENEURO.0242-18.2019)
Supplement: Extended Data Table 1-1 — Summary of statistical analyses by sex. Download Table 1-1, DOC file. [file sup_enu-eN-NWR-0242-18-s04.doc]

| **Extended Data Table 1-1. Summary of statistical analyses by sex** | | | | | | | |
| --- | --- | --- | --- | --- | --- | --- | --- |
| **Figures** | **Sex** | **N** | **Age** | **Type of test** | **Factor** | **Statistical value** | **p** |
| **3A** | F | rTgFKBP5 (1M-N=12; 3M-N=15, 4M- N=12; 6M-N=4); WT (1M-N=7; 3M-N=19, 4M- N=13; 6M-N=3) tTA (1M-N=3; 3M-N=6, 4M- N=12; 6M-N=4) | Varies | Two-way repeated-measures ANOVA with Bonferroni post-test | Genotype*Age | F = 1.266DFn = 6 | p=0.2803 |
|  |  |  |  |  | Genotype | F = 5.430; DFn = 2 | p=0.0058 |
|  |  |  |  |  | Age | F = 29.36 DFn = 3 | p<0.0001 |
|  | M | rTgFKBP5 (1M-N=4; 3M-N=11, 4M- N=9; 6M-N=3); WT (1M-N=12; 3M-N=14, 4M- N=14; 6M-N=3) tTA (1M-N=6; 3M-N=9, 4M- N=10; 6M-N=5) | Varies |  | Genotype*Age | F = 0.3316; DFn = 6 | p=0.9186 |
|  |  |  |  |  | Genotype | F = 1.008; DFn = 2 | p=0.3693 |
|  |  |  |  |  | Age | F = 35.76; DFn = 3 | p<0.0001 |
| **3B** | F | rTgFKBP5 (N=8), WT (N=12), tTA (N=9) | 3-month-old | One-way ANOVA with Tukey post-test | Genotype | F = 0.07656 | p=0.9265 |
|  | M | rTgFKBP5 (N=8), WT (N=10), tTA (N=11) |  |  | Genotype | F = 1.223 | p=0.3114 |
| **3C** | F | rTgFKBP5 (N=8), WT (N=12), tTA (N=8) | 3-month-old | One-way ANOVA with Tukey post-test | Genotype | F = 3.208 | p=0.0575 |
|  | M | rTgFKBP5 (N=8), WT (N=10), tTA (N=11) |  |  | Genotype | F = 1.584 | p=0.2243 |
| **3D** | F | rTgFKBP5 (N=8), WT (N=12), tTA (N=8) | 3-month-old | One-way ANOVA with Tukey post-test | Genotype | F = 12.89 | p=0.0001 |
|  | M | rTgFKBP5 (N=8), WT (N=10), tTA (N=11) |  |  | Genotype | F = 2.034 | p=0.1512 |
| **3E** | F | rTgFKBP5 (N=8), WT (N=12), tTA (N=9) | 3-month-old | One-way ANOVA with Tukey post-test | Genotype | F = 1.991 | p=0.1554 |
|  | M | rTgFKBP5 (N=8), WT (N=10), tTA (N=11) |  |  | Genotype | F = 2.908 | p=0.0740 |
| **3F** | F | rTgFKBP5 (N=8), WT (N=12), tTA (N=9) | 3-month-old | One-way ANOVA with Tukey post-test | Genotype | F = 6986 | p=0.5060 |
|  | M | rTgFKBP5 (N=8), WT (N=10), tTA (N=11) |  |  | Genotype | F = 0.8015 | p=0.4587 |
| **3G** | F | rTgFKBP5 (N=8), WT (N=10), tTA (N=11) | 3-month-old | One-way ANOVA with Tukey post-test | Genotype | F=7.128 | p=0.0036 |
|  | M | rTgFKBP5 (N=8), WT (N=10), tTA (N=11) |  |  | Genotype | F=0.5372 | p=0.5910 |
| **4A** | F | rTgFKBP5 (N=4), WT (N=5), tTA (N=5) | 4-6-month-old | Two-way repeated-measures ANOVA with Bonferroni post-test | Genotype*Training Day | F = 1.243; DFn = 6 | p=0.3034 |
|  |  |  |  |  | Genotype | F = 9.346; DFn = 2 | p=0.0004 |
|  |  |  |  |  | Training Day | F = 15.33; DFn = 3 | p<0.0001 |
|  | M | rTgFKBP5 (N=6), WT (N=5), tTA (N=5) |  |  | Genotype*Training Day | F = 0.7330; DFn = 6 | 0.6562 |
|  |  |  |  |  | Genotype | F = 2.562; DFn = 2 | 0.0868 |
|  |  |  |  |  | Training Day | F = 8.916; DFn = 3 | p<0.0001 |
| **4B** | F | rTgFKBP5 (N=4), WT (N=5), tTA (N=5) | 4-6-month-old | One-way ANOVA with Tukey post-test | Quadrant time | rTgFKBP5: F = 10.72; WT: F = 9.012; tTA: F = 2.815 | rTgFKBP5: p=0.001; WT: p=0.001; tTA: p=0.0725 |
|  | M | rTgFKBP5 (N=6), WT (N=5), tTA (N=5) |  |  | Quadrant time | rTgFKBP5: F = 3.127; WT: F = 63.03; tTA: F = 22.63 | rTgFKBP5: p=0.0487; WT: p<0.0001; tTA: p<0.0001 |
| **4C** | F | rTgFKBP5 (N=4), WT (N=5), tTA (N=5) | 4-6-month-old | Two-way repeated-measures ANOVA with Bonferroni post-test | Genotype*Training Day | F = 0.3830; DFn = 4 | p=0.8191 |
|  |  |  |  |  | Genotype | F = 9.821; DFn = 2 | p=0.0005 |
|  |  |  |  |  | Training Day | F = 8.376; DFn = 2 | p=0.0011 |
|  | M | rTgFKBP5 (N=6), WT (N=5), tTA (N=5) |  |  | Genotype*Training Day | F = 0.3677; DFn = 4 | p=0.8301 |
|  |  |  |  |  | Genotype | F = 2.580; DFn = 2 | p=0.0886 |
|  |  |  |  |  | Training Day | F = 3.054; DFn = 2 | p=0.0586 |
| **4D** | F | rTgFKBP5 (N=4), WT (N=5), tTA (N=5) | 4-6-month-old | One-way ANOVA with Tukey post-test | Quadrant time | rTgFKBP5: F = 2.743; WT: F = 10.59; tTA: F = 17.51 | rTgFKBP5: p=0.0894; WT: p=0.0004; tTA: p<0.0001 |
|  | M | rTgFKBP5 (N=6), WT (N=5), tTA (N=5) |  |  | Quadrant time | rTgFKBP5: F = 0.8729; WT: F = 24.65; tTA: F = 4.569 | rTgFKBP5: p=0.4716; WT: p<0.0001; tTA: p=0.017 |
